# Part III. Clinical Review and Hospital Reports

**Published:** 1833-10-01

**Authors:** 


### Clinical Review.

I. Clinical Reports, &c. By John Macfarlane, M.D. Senior Surgeon to the Royal Infirmary, &c.

### On Burns and Scalds.

We have drawn attention on several occasions of late to the subject of burns and scalds, because they are accidents that frequently occur, because we think they are often much mismanaged, and because there appears to be an idea, that there is something strange and mysterious in the action of heat on the living body. We need scarcely say that this idea is most absurd, and that cases of burn, when philosophically examined, present nothing mysterious or incomprehensible. In order to arrive at definite and rational methods of treatment in these or in any cases, we must study two things—the effects of the injury on the tissues exposed to it, and on the system; and the effects of remedies. The first is an exact investigation, the second an experimental one.

Unfortunately the investigation has been hitherto almost exclusively of the second description. Men have been blowing hot and cold, using stimulants and sedatives, lead and turpentine, but they have not studied accurately the varieties of lesion produced by heat, and the manner in which the lesion proves fatal. The consequence has been confusion, and instead of arriving at simplicity of reasoning and treatment, the profession has been absorbed in the abominable and unintelligible rigmarole of Dr. Kentish. This very instance is a triumphant answer to the opponents of morbid anatomy.

Let us reverse the order of proceeding hitherto adopted in the study of burns and scalds. Let us look at the fatal cases—let us mark the particular and general lesions—let us compare these lesions with the symptoms. Having done this, having learnt how burns prove fatal, we may rationally consider what means are adapted to the prevention of that result, and we enter on a course of experiments with remedies, knowing what to seek and what to dread. Any other method than this is false, fleeting, and empiric, and all who propose or sanction it, are embarking in a course of imposture or self-delusion.

The volume of clinical reports before us is valuable as a record of imperishable facts. We have turned to it before and shall turn to it again, for the author does not give us conclusions only, but the means which enabled him, and may enable others to form conclusions.—Such works are valuable in every age, for truth is still immutable. Will the theories of John Hunter outlive the facts of Morgagni?

Dr. Macfarlane relates eleven cases of burn, seven of which were fatal. For the reasons we have stated we will glance at the latter first, although the last in our author's category. We need scarcely say that burns may be fatal at three different periods—in the stage of collapse—in that of reaction—and at any subsequent epoch. It becomes of importance to ascertain the actual cause of death in all these stages. And first of that of collapse. It is manifest that this does not differ from the collapse occasioned by any powerful injurious impression or lesion, unless that lesion be attended with loss of blood. This is an important distinction, for collapse occasioned by that which occasions also a great loss of blood, must be very different in many respects from that of burn, where blood is not lost, but merely driven from the surface to the interior. It is probably on this account that consecutive visceral inflammations are, as we shall see, so frequent after burns. Dr. Macfarlane only gives one fatal case of collapse.

Case 1. *Severe Burn—Collapse fatal.*

M. C. æt. 2, admitted Nov. 16th, 1826. Six hours previously her clothes caught fire, and nearly the entire upper half of the body was scorched. The integuments of the neck, breast, abdomen, back, shoulders, and arms were brownish-coloured, hard, and completely charred. Warm turpentine was applied, but as it produced acute pain, it had to be removed, and the Carron oil was substituted. Brandy and ammonia were freely given, she was immersed in a warm bath, and all the usual methods for exciting reaction were employed, but without effect. The pulse was not to be felt, the skin was cold, the face pale and sharp, the pupils dilated and immoveable. Leeches were applied to the head, but the quantity of blood obtained was trifling; the stupor gradually increased, the respiration became laborious, and the child died comatose, twenty-eight hours after the injury.

On inspection, the vessels of the brain and its membranes were turgid with blood; there was an increased effusion of serum under the arachnoid and into the ventricles; the lungs were loaded with blood, as were the venæ cavæ and right auricle of the heart; but the mucous membrane of the lungs, stomach, and intestines was healthy.

The circumstances to be remarked in this case, with reference to structural alterations, are, the destruction of the integuments, which were killed by the action of the fire, and the congested condition of the lungs and brain. Now the latter is no trivial point. We were lately at a medical society, where a gentleman recommended that in cases of collapse from burn, the patient should be put under the influence of opium, and in point of fact, opium is very generally and very indiscriminately used in the collapse of burn. But we see that they die with congestion in the brain, and opium is known to give a tendency to this condition. Here then morbid anatomy shews, or seems to shew, that the administration of opium in such cases is unsafe. Collapse after burn is a state approaching to coma, and coma contra-indicates opium. The indication for opium is extreme restlessness or pain, but this is a condition opposed to what we may denominate comatose collapse. Dr. Macfarlane's observations on this collapse are judicious.

"When the shock is severe, the function of the brain and nervous system being in a great measure suspended, that derangement of the vascular and respiratory systems is produced, which forms so prominent a feature in this class of injuries. This state is characterized by a shrinking and coldness of the body, pale and contracted features, a rapid and feeble pulse, hurried respiration, and a greater or less degree of insensibility to pain, approaching sometimes to stupor. It may terminate fatally in a few hours, or continue for two or three days before death is produced, or reaction established; the severity and duration of the symptoms depending a good deal on the extent and situation of the injury, the cause by which it is produced, and the age of the patient.

The treatment usually adopted during the stage of prostration, consists in the free and frequent use of diffusible stimulants, with the application of heat to the trunk and extremities of the body;—diluted brandy, with small doses of the carbonate of ammonia and opium, will seldom fail, if carefully administered, in producing the desired reaction. Should we not succeed, however, as will sometimes happen, and there is a risk that the collapse may prove fatal, blood-letting may be adopted. In all such cases there must exist a considerable accumulation of blood in some of the internal organs, to which the imperfect action of the heart, and the feeble and thready pulse, may in part be referred: and should the patient be an adult, and of a robust habit, these symptoms may be beneficially acted upon by venesection, and thus reaction be more speedily established. I have only once had an opportunity of using the lancet during collapse from a severe burn. The usual stimulants had been exhibited for four hours, and failed; but after fourteen ounces of blood were detracted from the arm, a renewal of the stimuli proved more successful, and in less than an hour the heat was restored to the skin, and the pulse became full and bounding."

We should say that the best practical rule is this, to do no more in any way than is absolutely necessary. If we over stimulate, we produce visceral congestion or inflammation; if we deplete, we fear that we may do ultimate mischief, for as collapse occurs chiefly after serious and extensive destruction of parts, there must come a trying reparative process, when all the vigour of the constitution is wanted. We have found warmth applied to the surface a powerful and a harmless adjuvant, and we think that the surgeon should give as little brandy as possible. His object is, not to let the patient die of depression. If *he* produces reaction it is generally overdone. Coax Nature; that is all.

Children are often carried off by convulsions, within a few hours of the injury, or previous to the fifth day. When these appear early, or are rapidly fatal, slight traces of cerebral disorganization can be discovered. There is no difficulty in accounting for this, as convulsion is usually the consequence of slight structural alterations, or of merely functional derangements. Dr. Macfarlane gives a case in which convulsions occurred on the ninth day.

Case 2. *Scald—Convulsions fatal.*

E. S. æt. 3, scalded, Aug. 3d, 1831, on the back, buttocks, lower half of abdomen, and upper part of thighs. Admission four hours afterwards. The cuticle in some parts was raised into large vesications, and from others it was entirely abraded. Carded cotton was applied, and, as reaction had taken place, the bowels were freely opened, with calomel and castor oil, and afterwards small doses of an antimonial diaphoretic were given. The febrile excitement continued urgent, but the head did not appear to be affected until the 9th. At the hour of visit, the child was restless, and screamed occasionally; the eyes were suffused and irritable, the pupils dilated, the face flushed, and the tongue loaded.—*Leeches and cold applications to head.—Small doses of calomel.*—In the evening of the 10th, she was seized with convulsions, which continued with but little interruption during the whole night, and proved fatal on the following morning.

On inspection, the pia mater was unusually vascular, and there was an effusion of serum, with here and there a small patch of lymph between this membrane and the arachnoid. The vessels of the choroid plexus were turgid, and each lateral ventricle contained three drachms of turbid serum. The viscera of the thorax and abdomen were healthy.

Here there was arachnitis, a consequence of extensive scald. On inspection lymph was found between the arachnoid and pia mater. Now leeches were not applied till the 9th, when the pupils were dilated, and it is possible that as that dilatation was a symptom of pressure, and pressure a consequence of inflammation of some little duration, an earlier application of leeches and blisters might have been more successful. However this may be, practitioners may perceive that after burns and scalds they must keep their eyes open, and analyse the symptoms daily, that they may not let that pass, as a mere result of the injury, which is really a symptom of visceral inflammation. We will here introduce a case which occurred to ourselves, and is illustrative of the dangers of indiscriminate stimulation.

Case 3. *Scald—Cephalic Symptoms.*

On the 8th Sept. 1832, the child, æt. $1\frac{1}{2}$, of Mrs. B., Villiers Street, was severely scalded with boiling water on the chest, abdomen, and back. The child was immediately immersed in cold water. A medical gentlemen saw it one hour afterwards, and applied the turpentine liniment. We first saw the child on the 11th. It was feverish, with much irritability, thirst, and dark secretions from the bowels. What the treatment was we do not exactly know, as we merely saw the patient casually. We did not see the child again till the 13th, in the evening. There was then more pyrexia—the motions were very offensive—the aspect pallid—the pupils much contracted—no notice taken of surrounding objects. We found that the local treatment still consisted in terebinthinate applications—that the child was taking some bitter infusion—and that it had had three glasses of port wine that day.

We recommended abstinence from wine and the discontinuance of the bitter. On the next day we met the medical attendant in consultation. The child was now insensible—pupils firmly contracted—lower part of cornea covered with a film—face pallid—pulse frequent, jerky—one dark motion in the preceding night. We agreed on the following plan of treatment.

*Abstinence from wine and all stimulants—confinement to beef-tea and arrow-root—a powder of hyd. c̄ cret., pulv. rhei, and pulv. tragac. comp., twice or thrice daily. Locally, an absorbent powder, with the unguent. zinci c̄ plumbo. Castor oil. Warm baths.*

Before the evening three or four motions were procured, and the child was more conscious, and better in all respects. On the 15th, the head symptoms were nearly gone; the child was sensible, sat up, and took its nourishment well. There was now some diarrhœa

*Pulv. trag. comp., pulv. cret., comp. c̄ opio, pulv. rhei, stat.*

We need not pursue the details, suffice it to say that the bowels were soon regulated, and that on the 17th the child was able to take a little infusion of bark with infusion of rhubarb twice or thrice daily. The child got well with great rapidity.

We think there can be no question that, had the stimulating system been persevered in, this child would speedily have suffered from effusion in or on the brain, or inflammation of the membranes. But we must pass to other visceral affections besides those of the head.

"Besides the tendency to morbid changes in the brain, severe burns frequently prove fatal, by inducing inflammation of the serous or mucous tissues of the thorax or abdomen. Dupuytren, who appears to have investigated the pathology of this class of injuries with considerable attention, enumerates the following sympathetic or secondary lesions, as those which he has most frequently met with; inflammation of the intestinal, gastric, and pulmonary mucous membranes, of the serous membranes of the brain, thorax, and abdomen, and collections of blood and pus in the articulations of the burned extremities. I am not prepared to admit, that the mucous membrane of the digestive organs is more frequently the seat of the inflammation, than the serous membrane of the abdominal cavity, as Dupuytren seems to affirm. On the contrary, I am led to an opposite conclusion, by the dissections I have witnessed. These tend to corroborate the opinions of my friend Dr. Cumin, to whom the profession in this country is indebted for the earliest information on this important topic. He states, in his excellent practical paper on Burns, in the Edinburgh Medical and Surgical Journal for July 1823, that the mucous membranes suffer much less than those of the serous class, and that that cavity is most liable to be affected, the cutaneous surface of which is most extensively injured. I have also observed, that when this internal inflammation commences soon after the injury, and in an acute form, which it frequently does, the morbid changes it produces are usually confined to the serous tissues, but that the mucous membranes suffer more extensively when the disease is chronic, or does not appear till a more advanced period. The first case which I had an opportunity of inspecting confirmed this opinion."

Our experience, so far as it goes, agrees partly with M. Dupuytren's and partly with Dr. Macfarlane's. It tallies with M. Dupuytren's, in making affections of the mucous membrane more common than those of the serous; and with Dr. Macfarlane's, in respect to the periods at which these tissues are attacked. It is not, however, universally true that the mucous membranes only suffer at a late period, for we recently saw a girl die within ten days or a fortnight after a burn, in whom there were extensive ulcerations of the mucous membrane of the stomach and intestines. But to this case we will allude more particularly presently. And now of the cases of inflammation of the serous membranes.

Case 4. *Extensive Burn—fatal Pleurisy.*

A. M'L., æt. 34, admitted 8th June, 1831, having had his face, back, arms, and front of his chest burned seven hours previously, by an explosion of fire-damp in a coal mine. The cuticle was almost entirely abraded, and the cutis vera and subjacent parts were brownish-coloured, hard, and charred. He complained acutely of pain; he had slight shiverings; his extremities were cold, and his pulse was one hundred and eight, feeble. The excoriated parts were covered with lint, dipped in warm turpentine, and in an hour reaction was fairly established. On the following day, the chloride of lime was substituted for the terebinthine application, and the patient remained tolerably easy till the 12th, when, after a violent rigor, he was seized with acute pain in the left side of the chest, impeding inspiration, and accompanied with a troublesome, dry cough, great restlessness, and thirst; tongue thickly furred; pulse ninety-six, hard. There was a discharge of pus from the margins of the sloughs, which had begun to be detached. Was bled to twenty ounces; twenty-four leeches were applied to the pained part; the turpentine liniment was employed, and he was ordered three grains of calomel and one grain of opium every four hours. Notwithstanding a repetition of the blood-letting, and a continuance of the other antiphlogistic means, with the use of smart purgatives, and nauseating doses of emetic tartar, the symptoms were not relieved in the slightest degree. He was bled, leeched, ordered calomel and opium, and subsequently tartar-emetic, &c. but without avail. The symptoms increased, delirium supervened, and he died on the 16th, eight days after the burn, and four from the commencement of the thoracic symptoms.

On inspection, the left side of the thorax contained a pound of sero-purulent fluid, in which flakes of lymph were observed floating; the lung was considerably compressed, and its investing pleura, as well as that portion of the membrane lining the ribs, was of a deep-red colour, and covered here and there with thick patches of shaggy lymph. The inner surface of the pericardium was inflamed, but there was no effusion into its cavity. The mucous membrane of the lungs, stomach, and intestines, was natural.

Case 5. *Burn—fatal Inflammation and Gangrene of the Intestines.*

J. G., æt. eight, was admitted on the 25th of January, 1827, two hours after his clothes had caught fire. The integuments covering the abdomen, pubes, and thighs, were abraded, hard, and presented a greyish-brown leathery appearance. The pulse was so rapid and feeble, as not to be counted; the body was cold, and he had rigors. Warm turpentine was applied, and in a few hours reaction was established by hot flasks to the extremities, and the exhibition of the usual stimulants, after which the bowels were opened by castor oil. On the evening of the 27th, there was a return of the coldness, and tendency to rigor; the injured parts were hard and tense; the respiration quick and laborious; the pulse rapid and feeble; the countenance pale and anxious. He was immersed for a few minutes in a warm bath, and ordered small doses of calomel. On the 31st, there was great restlessness, alternating with fits of drowsiness, approaching to coma; the abdomen was slightly tympanitic, but without pain on pressure; the bowels were loose; there was no vomiting. He expired on the evening of the 3d of February.

On inspection, the peritoneum covering the intestines and abdominal parietes was extensively inflamed, and two gangrenous openings were discovered in the duodenum, near its termination. One of these admitted the finger, and from both a free discharge of feces had taken place into the upper part of the cavity of the abdomen, where it was confined by recent adhesions of the intestines. From the lower part of the cavity, near the pelvis, six ounces of a bloody-coloured viscid fluid were removed. There was a considerable quantity of blood effused around the capsule of Glisson, and between the layers of the omentum. The mucous membrane of the small intestines showed marks of acute inflammation, but only a slight tendency to ulceration was perceptible. The brain was in a state of congestion, and the ventricles contained rather more serum than natural.

Dr. M'Farlane observes that burns, occurring during the period of utero-gestation, are apt to excite premature labour, with or without the presence of abdominal inflammation. In one case which occurred to our author, repeated and profuse uterine hæmorrhage occurred, apparently from detachment of a portion of the placenta. The patient would have sunk, had not artificial delivery been resorted to. Our author details in full another case of burn during pregnancy. The patient, æt. 44, was in the eighth month; the abdomen, pubes, and thighs were severely scalded. On the 4th day she had a rigor, followed by acute pain in the right inguinal region, which speedily became diffused over the abdomen, with alarming symptoms. By bleeding to 58 ozs. calomel and opium, &c. the symptoms were gradually subdued. On the 8th day after the reception of the burn, pains came on, and, after a severe labour of seven hours, she was delivered of a dead child. What is curious, there were several bullæ on the child's abdomen and thorax, although there was no appearance of commencing putrefaction. This patient slowly recovered.

So much for the cases of serous inflammation. Our readers will perceive that, though analogous in some respects with the secondary serous inflammations, after injuries and operations, they are, if we may be allowed to build any thing on the last case, less inevitably fatal. We must, however, wait for more facts before we decide. We proceed to those cases in which the mucous membrane was affected, and we do this the more willingly, as we believe that many false notions prevail on this head.

CASE 6.—*Burn—fatal Ulceration of the Mucous Membrane of the Intestines.*

M. M. æt. 5, admitted Feb. 23d, 1826, with extensive burn of the abdomen and back, from her clothes having caught fire. On May 1st, more than one-half of the ulcerated surface was healed, but she was emaciated, and the health impaired. On June 1st there was only, on the hypogastrium, a sore an inch broad and three inches long. This part ceased to heal, and the granulations became pale-coloured, and gelatinous. No febrile excitement, however, took place; the appetite continued good; the bowels regular; and the tongue clean. On the 19th, after a slight rigor, she complained of pain in the abdomen, midway between the pubes and umbilicus, which was aggravated by pressure; the pulse was quickened, the heat of skin increased, the tongue furred, and the bowels loose and irritable. Leeches and fomentations were applied, and she had a small dose of *oleum ricini,* followed by *calomel* and *creta,* a *warm bath,* &c.

On the 20th she had another rigor, followed by increased febrile disturbance, diffuse pain, and slight tympanitic swelling of the belly; a very rapid and feeble pulse, urgent thirst, vomiting, diarrhœa, and an anxious collapsed countenance. In the course of a single night, the granulations were absorbed from the unhealed sore, and a hollow cavity produced, which was pale-coloured, smooth, glassy, and dry. A considerable portion of the new cicatrix re-ulcerated. She became gradually more and more exhausted, and expired on the evening of the 28th, about four months after the reception of the burn.

On inspection, there was very slight peritoneal inflammation. The mucous membrane of the lower half of the duodenum, and of the jejunum and ileum was very vascular, infiltrated both with blood and serum, and extensively ulcerated.

Dr. M'Farlane remarks, that it is difficult to account for the peritoneal inflammation at so late a period. It seems to us that the peritoneal inflammation was rather dependant on the affection of the mucous membrane, than immediately on the burn. Every one accustomed to the examination of bodies is aware that peritonitis is a very common attendant on ulcers of the mucous membrane in fevers, &c. and the relations of the tissues are sufficient to account for the fact. The following remarks appear to us to be more pertinent.

"When, from the extent and severity of the burn, a high degree of constitutional irritation has been excited, and so long maintained as to have impaired the health, and produced considerable emaciation, it not unfrequently happens that chronic disease of the alimentary mucous membrane supervenes. This cannot always be attributed to the direct influence of the local affection, but it must sometimes depend on the continued constitutional excitement which the injury has produced. There are many chronic diseases which ultimately lead, in this way, to an inflamed, softened, and ulcerated state of the alimentary mucous tissue; and where the secondary disease is to be referred to the injurious excitement which has been so long maintained."

There can be no question that fever, however induced, will, if long kept up, induce local inflammations and alterations, in organs or tissues remote from that where the origin of the fever exists. Thus, the hectic occasioned by disease of the foot becomes the cause of disease in the lungs—or, if produced by disease in the lungs, it gives rise to ulcerations of the bowels—the symptomatic fever of a suppurating limb, after compound fracture, is the precursor of visceral inflammations or deposites; and so on. It is highly necessary to attend to these facts, even if the principle we have founded on them is disputed, for, practically speaking, we have always reason to dread the existence of such fever, and should endeavour to get rid of it. This can only be done, of course, by removing the cause.

Case 7.—*Severe Burn—fatal Ulceration of the Intestines.*

M. M'D. æt. 18, admitted Jan. 2d, 1832, having had her clothes set on fire a few hours previously. The back, nates, perinæum, labia, and thighs were vesicated, denuded of cuticle, and in some places charred; the pulse was rapid and indistinct, the thirst urgent, the body cold, and the restlessness great. The parts were put up in cotton, and when the febrile excitement commenced, a purgative was ordered. The pulse continued rapid; she was obliged to lie constantly on her face; and, as her habits were most filthy, and she voided her urine and feces in bed, the sores healed very slowly. From these causes, while the other parts had nearly cicatrized, the right thigh became sloughy, the appetite impaired, and the bowels irritable. Her strength continued to diminish daily; diarrhœa set in, with occasional vomiting; the countenance became Hippocratic, and she expired on the 12th of February.

On inspection, there was found superficial and deep-seated congestion of the brain, with serous effusion under the arachnoid, and into the ventricles. The liver was immensely enlarged and tuberculated; the mucous membrane of the small intestines was injected, tumid, and ecchymosed; the ileo-cœcal valve was in a state of ulceration, as were occasional portions of the colon and rectum.

Dr. M'F. imagines that, by strict attention to the state of the bowels and the skin, he has occasionally succeeded in warding off these attacks of internal inflammation, even when there were premonitory symptoms. We would beg to draw attention to the affection of the mucous membrane of the intestines after burns. Nothing is more common than to hear two remarks, one of which is imperfect—the other, for the most part, erroneous. The remarks to which we allude are these:—First, that diarrhœa is a consequence of burn, and often a good sign; and, secondly, that patients die of exhaustion from burn, meaning, we suppose, that they die because they cannot live.

With respect to the observation on diarrhœa, it is, as we have said, imperfect. The diarrhœa is a sign of that condition of the intestinal mucous membrane, which, if neglected or maltreated, or even managed with the utmost care, is the prelude to the ulcerative action. When that diarrhœa appears, the judicious surgeon will regard it with a jealous eye, and adopt the treatment applicable to chronic muco-enteritis. A

patient is usually constipated soon after the infliction of a burn, and the constipation as usually gives way, in three or four days' to diarrhœa. We have seen a most uncontrollable looseness brought on by the impatience of the surgeon, in throwing in powerful cathartics during the torpid condition of the bowels alluded to. Let him wait till Nature is inclined to work, and let him rather entice her by mild aperients or injections, than force her by drastic purgatives. He will usually have purgation enough before he has done with the case.

We have said that the remark on patients dying of exhaustion is probably erroneous. It is possible, no doubt, that a patient may have such extensive ulceration of the skin as to prove, in itself, a cause of death. But this is not generally the case, and in this, as in other instances, death is produced by visceral disease, usually ulceration of the mucous membrane. The surgeon who believes his patient is dying of exhaustion, will probably order additional support, if not stimulus, as his patient seems more exhausted. The surgeon who is aware that visceral disease is being established, will know that such treatment is certain to be destructive. He will support without stimulating, and will attend to the secondary alteration.

The majority of those whom we have seen die, at any distance of time after the infliction of the burn, have certainly sunk from ulceration of the intestinal mucous membrane. We lately saw a remarkable instance of perforation of the stomach, in consequence of an ulcer which began in its mucous coat. There had been hæmatemesis and voiding of blood by stool before death, which took place rather suddenly; the abdomen was tender on pressure. On examination, there was slight peritonitis, and the perforation we have mentioned. There were various other ulcers in the stomach, and ulcers, also, at the termination of the ileum. The patient died between the second and third week. To this case we alluded in a preceding part of this article. In another case of death after burn, we saw similar ulcerations of the stomach, unattended with perforation.

A case of tetanus after burn is related in the Glasgow Medical Journal, for April of the present year. Tetanus, being a comparatively accidental consequence of any injury, is not possessed of much interest here. We notice the case for the sake of the dissection. The patient was an epileptic, and the part chiefly injured was the right upper extremity. Trismus appeared on the 12th day, and death occurred on the 17th.

On dissection, the anterior roots of the fourth and fifth cervical nerves of the right side were of a bright red colour, compared with those above and below. Two of the middle nerves of the brachial plexus of the right side were of a dirty yellow colour, perfectly distinct from the characteristic whiteness of the phrenic and vagus of the same side. This appearance commenced about an inch above the clavicle, and was traceable downwards into the axilla. The sheaths of these nerves, in the same situation and to the same extent as their discolouration, were sensibly reddened, and numerous distended red vessels were seen in their texture. The median and ulnar nerves were of the same yellow colour, in the greater part of their course through the diseased parts, and their neurilema was injected for two inches below their origins from the brachial plexus.

This article has, so far, been confined to the secondary consequences of burn, to the morbid anatomy, to speak improperly, of the lesion, and to those practical deductions which may obviously be drawn from the dead body. The remainder, and it will be short, applies to the treatment of burns and scalds, independently of visceral complications. We have lately dedicated some pages to this part of the subject, and shall not repeat what we have already said. We refer our readers to our notice of Mr. Earle's two lectures on burns.

The treatment of burns and scalds recommended by Dr. M'Farlane is this —when the cuticle is extensively vesicated, and the surrounding integuments inflamed, the immediate application of finely-carded cotton, the vesications having been previously punctured. Several layers of the cotton should be applied, and retained in close contact by means of a roller. Two cases are related, illustrative of the good effects of this treatment in superficial burns, in which Dr. M'F. observes that its advantages are most conspicuous. Dr. M'F. however, has found it very effectual in cases where the disorganization is deeper.

A case is detailed, in which the application of cotton, combined with generous diet and wine, effected a cure.

"When the burn has penetrated deeply, and the eschar is still adherent, I prefer applying the turpentine liniment, or a solution of the chloride of lime, to the use of cotton. I seldom have recourse to poultices for the purpose of facilitating the detachment of the charred integuments, these warm and emollient applications, although decidedly useful in accelerating the ulcerative disunion of the sound and gangrenous parts, tend to induce exuberant and spongy granulations, and of course to establish that kind of unhealthy action which forms so serious an obstacle to cicatrization. When the injury is confined to the trunk of the body, the turpentine liniment is employed; but when the extremities are implicated, I have found the chloride of lime solution infinitely preferable. When these applications are had recourse to, several layers of fleece cotton are laid over them, and the dressings are seldom removed until the sloughs have become detached, and a change of the treatment is required."

Dr. M'F. however, has found the cotton injurious in some cases, and these he mentions generally in the following summary.

"In three cases of simple vesicated burns, the application of carded cotton, soon after the injury, was productive of such acute and continued pain, that it had to be removed, and a different remedy employed. In two cases of superficial burns, where the cutis vera was in a state of ash-grey ulceration, and the surrounding integuments presented a bright red colour, the local and constitutional excitement was so great, as to render the discontinuance of this application absolutely necessary. In four cases, during warm weather, the discharge was so profuse, the fetor so intolerable, and the generation of maggots so abundant and annoying, as to require a daily change of the dressing; and besides these inconveniences, the health of the patients became greatly impaired, the appetite diminished, and, in one of the cases, incessant vomiting and diarrhœa were excited. When these untoward circumstances manifest themselves, and especially when the injury is confined to the limbs, I have found the application of a solution of the chloride of lime exceedingly beneficial. In one case, where the front of the chest and abdomen was scalded with boiling water, lint, moistened in a solution of the chloride (containing gr. ij. of the salt to an ounce of water) was applied and kept wet for ten days before it was removed, when nearly two-thirds of the abraded surface were cicatrized. The second dressing was removed four days after, when the cure was complete; acute pleuritis however occurred, and required free bleeding, purging, and the use of emetic tartar in solution. In another case, both lower extremities were scorched by the clothes catching fire; and, of course, the injury extended deeper than in the former one. The same treatment was pursued, although, from the nature and situation of the injury, and the advanced age of the patient (fifty-nine years), the cure was more protracted. The first dressing was removed on the twelfth day, when the greater part of the leathery slough, produced by the destruction of the cellular texture, was found separated, exposing a florid granulating surface. The lotion was re-applied, and continued nearly three weeks longer, before cicatrization was completed.

I consider this a very excellent application to burns; it stimulates moderately, destroys fetor, and, when surrounded with oiled silk, it maintains a degree of heat and moisture, favourable to the separation of sloughs, and to the subsequent cure. In my estimation, however, no small share of the credit is due to the mode of its employment. Instead of removing the dressings daily, and thus exposing an extensive and inflamed surface to various sources of irritation, they are allowed to remain for several days, care being taken to keep the parts constantly moist, by the assiduous application of the lotion. The injured parts are thus preserved in a state of perfect quiescence, and all external interruptions to the healing process are avoided. Before the lotion is applied, if the vesications are large and tense, it is necessary to evacuate the serum by a number of small punctures, allowing the detached cuticle to remain, which forms a useful covering to the tender surface, and does not interfere with the subsequent cicatrization. Should the burn be extensive, and confined to the trunk of the body, the constant retention of wet dressings has sometimes chilled the patient, and given rise to internal inflammation; in such cases, the cotton is the preferable application, but when the extremities are involved, I have found the solution of the chloride of lime on many occasions eminently successful."

A case of burn of the face, cured by chloride of lime, is detailed. It presents no features of interest. We have now concluded that part of Dr. Macfarlane's book which relates to the subject of burns, and we make no apology for the length of the notice. We are satisfied that practitioners are too generally unacquainted with the true pathology, and, consequently, correct treatment, of these injuries.

---

### II. CLINICAL LECTURES, BY DR. ROOTS.*

We have felt much pleased with many of the observations delivered by this active and talented physician in his clinical lectures, published in our weekly contemporary, the London Medical and Surgical Journal. We have no intention of analyzing these lectures, but we merely notice a few of the more interesting facts related by the lecturer, and made by him the peg on which his remarks are hung.

### *Fever.*

*Case.* S. Green, æt. 51, was admitted into St. Thomas's Hospital, March 28th, 1833. She had been nurse, and had been ill for a week. She was attacked with shivering, followed by pain of the head and back, with feeling of great weakness, heat of skin, great thirst, and dryness of the mouth; inability to sleep, and great restlessness during the night; her tongue was white and dry. She complained, too, of some cough; her bowels, she said were quite open, but the motions were very dark-coloured.

There being no evidence of particular or prominent lesions, Dr. R. merely prescribed five grains of the hyd. c̄ cret. thrice daily—castor oil—tepid washing—and slops. The alvine secretions soon became natural; medicine was then discontinued, she progressively improved, and was discharged on the 11th April.

Dr. R. observes that he has found mercury useful in fever, in proportion as it is accompanied with inflammation or congestion, or derangement of the secretions. When these indications pass away, the mercury should be discontinued. Of mercurial preparations, he prefers the hyd. c̄ cretâ.

### *Porrigo.*

In a case of *porrigo scutulata,* the following treatment was adopted. The head was shaved every week; and, as the patches were not irritable, tar ointment was applied night and morning, the head being properly washed every morning before the application of the ointment. As her health was very good, she took no medicine during the first sixteen days; but as the bowels then became sluggish, she was ordered gr. x. of the pulv. rhei c̄ hydrargyro, every other morning. The eruption had very much improved, but, at the expiration of four weeks, it appeared

* Lond. Med. and Surg. Journal. June 29th, 1833.

stationary, indolent, and to want a more stimulating application than the tar-ointment alone. The tar ointment was now used, in combination with the ung. sulph. comp. in the proportion of ℥j. of the former to ʒj. of the latter. This was applied for two days, when much irritation was induced; it was then omitted, and zinc ointment substituted; the irritation subsided, no fresh pustules came out, the redness of the patches diminished, and she was dismissed well in less than two months from her admission.

Dr. R. observes that no one plan of treatment will succeed in all cases of porrigo. We may say the same of most cutaneous complaints, especially the pustular and vesicular. If there is much irritation or inflammation, sedative applications are useful. A vast deal of mischief is daily done by inattention to this circumstance; and many a time and oft have we seen poor wretches tormented, for month after month, by nitric acid, caustic, and other highly irritating substances. We have frequently checked porrigo in its commencement, by careful ablution with warm soap and water, shaving of the head, the application of bread poultices, made with Goulard water, and attention to the state of the digestive organs.

When the inflammation has become indolent, stimulants are necessary. Dr. R. recommends the unguent. picis, diluted with the ung. zinci, in the proportion of one part of the former to four or five of the latter, increasing according to circumstances the quantity of the former. Sometimes it is necessary to combine tar ointment with some of the ung. sulph. comp. or with the ung. hyd. nitratis. If these fail, Dr. R. mentions a strong solution of the nitrate of silver—blisters—pyroligneous acid, added to a bread and water poultice. Children should be separated, and the same combs, &c. not used indiscriminately.

### *Acute Rheumatism.*

Passing over some cases of different kinds, and one of acute rheumatism, of a rather interesting character, we will give Dr. Roots' ideas and experience on the treatment of rheumatism. After commenting on the bark treatment, which he has not seen successful, he goes on to state that he has found moderate bleeding, in the early stage, beneficial, and never had reason to believe that pericarditis was a consequence of it; indeed, his practice has led him to the opposite conclusion. He then proceeds to observe, with respect to colchicum, that it has proved, in his opinion, one of the most useful remedies in the treatment of rheumatism.

"Many, I believe (says Dr. R.), consider that it possesses specific powers over the disease; I do not think so; I have never seen it exercise any control over the disease unless it purged or vomited, and have always seen it prove most useful when it purged pretty actively. You will find, too, many other hydragogue cathartics, as they are termed, equally useful in checking rheumatic inflammation; elaterium, for example, and camboge. More than ten years ago, I treated several cases of acute rheumatism in St. Pancras Infirmary, by bleeding, and a grain of elaterium, given every morning for three or four days, and I found the cases get well quite as quickly as those treated with colchicum, but as the elaterium is less manageable, more likely to distress the stomach and excite vomiting than the colchicum, I certainly prefer the latter; I believe that its good effects in rheumatism are ascribable to its action as a counter-irritant on the mucous membrane of the alimentary canal, exciting, as it were temporarily, disease in a tissue of the body, different from that which is the seat of rheumatic inflammation, and at the same time diminishing the general excitement of the system through the evacuations which it produces, as I believe it to be much more useful when it purges, than when it excites vomiting only; I am, as you well know, in the habit of generally combining it with an alkali, or alkaline earth, as the carbonate of magnesia, which combination tends to diminish its emetic effect, and at the same time increases its purgative action; if this, however, is not sufficient, and it often is not, then its emetic property may be still further controlled by adding a minim or two of hydrocyanic acid to each dose, for the knowledge of which valuable fact I am indebted to Dr. Elliotson. But useful as colchicum is generally in the treatment of rheumatism, you will not unfrequently meet with cases in which the disease mocks its employment, and this is more especially the case where the inflammation is confined to bursal or ligamentous tissues, here you will find local depletion by leeches, cold application, and mercury, do far more good than colchicum."

In this estimate of colchium we coincide with Dr. Roots. We are in the habit of employing it extensively in many affections, and have ever observed, as Dr. R. has done, that it has done good in proportion as it has purged or vomited. We do not think so much of the combination with magnesia, excepting in cases of gout, as Dr. Roots appears to do. We usually combine it with antimony and with sulphate of magnesia, in saline draughts, and although we often give doses of half a drachm every four hours, we have not yet had reason to repent our boldness. Of course the patient must be watched, and of course, also, the remedy must be suited to the stage of the disease and the powers of the patient. It is only when there is much inflammatory action that we would think of pushing antimony and colchicum.

### *Hepatic Abscess.*

Two cases of this description have been related—one by Dr. Roots in his Clinical Lectures—the other by Dr. Stokes in his. We will give a short notice of each. And first of the case of Dr. Roots.

*Case** 1. G. Scully, æt. 28, a carter, was admitted into St. Thomas's Hospital, Jan. 25, 1833. He complained of acute pain in the situation of the cæcum, increased on pressure, and extending slightly upwards in the course of the ascending portion of the colon—no perceptible hardness—inability to lie upon the right side—tongue white in the centre, red at the point and edges—bowels loosely opened once daily, with some griping—pulse 100, small, rather sharp—emaciation. He had been ill one month. He was thought to be labouring under inflammation of the cæcum, and was treated thus. Twenty-four leeches were ordered to be applied over the painful part; half an ounce of castor oil was directed to be given directly, and five grains of the hydrargyri cum creta every six hours, his diet to be milk and arrowroot. Now, this plan of treatment was continued for about a fortnight, the leeches being repeated nearly every day; the pain diminished, and on the 8th of February he was quite free from pain, with or without pressure.

The mouth was now slightly affected by the mercury, and it was discontinued. He remained free from pain from Feb. 8 to Feb. 16, and was able to get up. He committed an excess in diet, and on the latter day he was confined to bed, with return of pain and pyrexia. He was again ordered leeches and castor oil, but on the 27th, there being still considerable pain, the mercury was resumed. After a week it was again discontinued, in consequence of diarrhœa, with tympanitic abdomen. The diarrhœa was checked by starch injections with laudanum, when mercury was resumed, in combination with opium, leeches being still continued.

Distinct hardness was now felt, extending from the region of the cæcum to the right hypochondrium, and from that to the left. The resonance on percussion was dull. Poultices were applied. The cough now became more frequent, the respiration more hurried, the resonance of the inferior portion of the right side of the chest dull, and the respiratory murmur indistinguishable below the fifth or sixth rib. On the 23d March, circumscribed fluctuation was evident in the right hypochondrium. The mercury, &c. were omitted. On the 26th, Mr. Green punctured the ab-

* Lond. Med. and Surg. Journal, July 27.

scess, and let out four pints and a half of the most horribly offensive greenish-coloured pus. One week after this the patient died.

On dissection the right lobe of the liver was found to be the site of the abscess. This was contained in two large cavities, communicating with each other by a sort of valvular opening. Adhesion of the surface of the liver to the abdominal parietes had taken place, and there the trocar had been introduced. Adhesion had also taken place between a great portion of the convex surface of the liver and the diaphragm, and between the latter muscle and the right lung, the latter being pushed up as high as the fourth rib by the growth and pressure of the abscess. The lung itself was considerably condensed, but the surface of the liver and the diaphragm were so attenuated, that probably they would soon have given way, and the abscess would then have burst into the cavity of the thorax. The cæcum shewed traces of inflammation, and even of ulceration.

Dr. Roots observes that he has not seen above six or seven cases of abscess of the liver. The first was treated by Dr. Haviland, of Cambridge. An opening was made with a trocar, and pus and hydatids discharged. The patient, a female, recovered, and died three or four years afterwards of phthisis, as Dr. Roots believes.

In the preceding case the hepatic abscess had almost emptied itself into the lung. In the following, that event would seem to have taken place.

*Case* 2.* The patient entered the Meath Hospital, with cough, pain in the right side, and copious expectoration, slightly tinged with blood. On examination a tumour was found in the region of the liver, with corresponding dulness on percussion. On investigation it appeared that the history of the case was this. Six weeks previously the man was attacked with severe pain in the region of the liver. At the end of three weeks, without any previous symptom of disease of the lung, he was seized with a sudden fit of coughing, and expectorated a vast quantity of pus, and it was found that, after the cough and expectoration had lasted for some time, *the hepatic tumour decreased considerably in size.*

It was concluded by Dr. Stokes and all concerned, from these circumstances, that the case was one of hepatic abscess, perforating the diaphragm and opening into the substance of the lung. We extract the following remarks, premising one of Dr. Graves', that the opening of hepatic abscess into the lung is one of the most favourable terminations of the disease, one most frequently followed by recovery. Dr. G. proceeds thus.

"A great number of such cases have been observed, but very few of the patients have undergone examination with the stethoscope; I have related one in the London Cyclopædia of Practical Medicine. In the present case, however, besides the symptoms before mentioned, our opinion was borne out by other considerations. The intercostal spaces were not obliterated; the hepatic tumour, also, was still evident. Now, this might be considered an enlarged or a displaced liver; if displaced it must be in consequence of a vast empyema of the same side: but of the existence of this there was no indication. You remember that, while on the subject of empyema, I remarked, that where we had dulness of sound and absence of the respiratory murmur in the lower and posterior part of the lung from the pressure of an enlarged liver, when we make the patient take a deep inspiration, the respiration becomes audible. This did not occur in the present case. It is probable that, in a case where hepatic abscess bursts into the lungs, the adhesions which are formed prevent the descent of the lung on that side, and to this source we are to attribute the defective nature of the diagnosis. I recollect having seen a case of abscess on the liver with a double opening. The woman had pain and swelling of the right hypochondrium while she was here, and some time af-

* London Med. and Surg. Journal, Aug. 3, 1833.

terwards an evident pointing. She left the hospital about this time, but returned shortly afterwards with a large swelling, like an anthrax, on her side. This was opened by incision, and a vast quantity of pus gushed out. On dressing this one morning, a quantity of air escaped, and on introducing a probe, it seemed to pass in the direction of the diaphragm. We were therefore led to conclude that the hepatic abscess had not only an external opening, but had also passed through the diaphragm and the substance of the lung. On examination, after death, we found that the abscess was not in the substance of the liver, but external to it, and that, from this situation, it had passed through the diaphragm and the substance of the lung. With respect to the case at present before us, all we can do is, to support the man's strength, and produce resolution of the hepatic tumour; in fact, we must treat it as a case of chronic hepatitis."

---

### III. Clinical Lecture of M. Dupuytren on Fissure of the Anus.

[Leçons Orales. Tome troisième.]

Fissure of the anus, though not commonly a dangerous, is an extremely painful affection. The patient usually complains of excessive torture, comparing it to the sensation produced by a red-hot iron introduced into the rectum. The expulsion of the fæces is productive of so much suffering, that he will even abstain from food to avoid it.

Fissure of the anus consists in a long superficial ulceration, developed near the margin of the anus, in the radiated folds of the mucous membrane. On separating the sides of the anus, and making the patient force down, a narrow chink is perceived; its surface red, its edges tumid and callous. The introduction of the finger is often necessary to ascertain the extent to which it passes. It is situated more frequently on the lateral or posterior, than on the anterior face of the anus, and seldom attacks its whole circumference.

The severity of the affection depends on the spasmodic contraction of the sphincter, which occurs on the contact of the smallest foreign body.

The causes of the complaint are numerous. Constipation—the passage of hardened fæces, distending the gut and lacerating the mucous membrane—the clumsy exhibition of lavements, especially when metallic pipes are employed—hæmorrhoidal complaints—the venereal virus.

The inefficacy of local applications has led to the employment of two measures, and of them almost alone—division of the sphincter, and cauterization with the nitrate of silver. Both are so painful, though generally successful, that a less severe method is a desideratum. M. Dupuytren has employed one, which, although not constantly effectual, is sufficiently fortunate to make it worthy of trial, prior to resorting to an operation.

Spasmodic contraction of the sphincter is the essence of the malady; the ulceration is only a secondary phenomenon. The object of M. Dupuytren's treatment is to moderate this spasm, which he does, by the introduction within the anus of the extract of belladonna. The composition of the ointment he employs is as follows:—

℞. Axungiæ .......... ps. vi.

Extracti belladonnæ .

Plumb. acet..... āā pm. j.

M.

A tent, of a moderate size, is smeared with this, and passed into the rectum. The size of the tent is gradually increased. The constant employment of the ointment for some days frequently occasions complete relief.

Three cases are related.

*Case* 1. A young woman was admitted into the Hôtel Dieu. She had been confined four months previously, and had experienced, for some weeks, severe pains in the anus, aggravated each time she went to stool, especially if the fæces were bulky and hard. At the commencement of the complaint, the paroxysms of pain had only lasted for a few minutes, but they became more and more prolonged, and at the period of her admission would continue for several hours.

On examining the anus and drawing out the lower extremity of the gut, a very superficial fissure was discovered. The contraction of the sphincter was such that the little finger could with difficulty be introduced, and it occasioned much suffering. The ointment above stated was smeared on a tent of lint and passed into the gut several times daily. In fifteen days the patient was completely cured.

Ulcerated fissures are met with in three situations, below the sphincter, above it, and within its embrace.

Those below the sphincter occupy only the cutaneous texture, and do not involve the mucous; they occasion more or less pruritus, but produce no pain in defæcation, nor contraction of the sphincter; they are, therefore, not very painful. Most commonly they are produced by the venereal virus.* Those seated above the sphincter affect the mucous membrane, and require the speculum to disclose them. On passing the finger into the gut, there is felt in the situation of the fissures a hard knotted cord, pressure on which occasioned acute pain. They occasion tenesmus on going to stool, relieved after its excretion. The fæces are smeared with puriform mucus, and with blood on the side of the fissures. They are commonly due to the ulceration of internal hæmorrhoids, occasioned by the passage of hardened fæcal matters. Those fissures situated within the sphincter are the most severe affection of the whole, and are accompanied with the spasmodic contraction of the sphincter.

Fissures of the first and second description are usually cured without an operation, by the application of ointments, injections, &c. But in the last mentioned kind of fissure the most speedy and most effectual treatment consists in the operation first recommended by M. Boyer.

*Case* 2. A man, 28 or 30 years of age, had experienced for more than four months pains in the fundament aggravated by defæcation. Latterly his suffering had become almost insupportable; it was worse after the excretion of the stool, and lasted for four or five hours. He had employed without benefit a variety of treatment, before he was received into the Hôtel Dieu.

There were a small excrescence around the anus, spasmodic contraction of the sphincter, and a fissure on the left side. The excrescence was removed by scissors, and the ulcer incised, after which lint, smeared with ointment, was introduced into the rectum, and kept between the lips of the incision.

M. Dupuytren remarked on this case, that the division of the ulcer itself is preferable to an incision made at a little distance from it. But when the ulcer is on the anterior wall of the gut, towards the urethra of the man or the vagina of the woman, the incision should not be through it.

In old cases the local malady may be attended with severe constitutional disturbance.

*Case* 3. Angelica Delahaye, æt. 24, of good constitution, and regular, had had several children. She was admitted into the Hôtel Dieu with several fissures of the anus, and with excrescences. The anus was contracted. She rarely went to stool, and when she did so, she had dreadful suffering, prolonged for several hours after the evacuation.—Most commonly the fæces were mixed with a great quantity of blood and mucus. The patient's health was impaired—the surface of the body was pallid and puffy, and she frequently had fever. The disease had existed for several years, was attributable to no known cause, had been attended with little inconvenience at first, and had increased by slow degrees.

---

* Those depending on the venereal virus, usually extending from the perinæum to the verge of the anus, and frequently attended with excrescences of cutis, which indeed are their seat, are generally curable without difficulty by the application of black-wash in the first instance, followed after a day or two by the oxymuriate lotion, in the proportion of half a grain to the ounce of water.—*Eds.*

After preparatory treatment for a few days, M. Dupuytren excised the excrescence and divided the fissures with a probe-pointed bistoury; several incisions were necessary. A tent of lint, of the size of the finger, was introduced into the anus to prevent the sides of the incisions from uniting.

On the same day the patient had a copious stool, accompanied by considerable loss of blood, but unattended with the usual severity of pain. A new tent was instantly replaced in the rectum. The dressing was re-applied every day and every time the patient had a stool. In twenty-two days after the operation she left the hospital cured.

The foregoing clinical lecture is interesting in connexion with the notice of Mr. Mayo's work, at the commencement of this number.

---

### IV. DR. LATHAM'S CLINICAL REMARKS ON THE DURATION OF FEVERS.

[Bartholomew's Hospital.]

ALL the good old notions in medicine are fast disappearing before the modern rage for precise induction and morbid anatomy. The doctrine of critical days in fevers has been for many years consigned to the tomb. But the belief in specific periods of duration has survived, and old practitioners of the present time yet talk of fevers of twenty-one and of fourteen days, as if their existence were beyond possibility of doubt. The able and the intelligent smile at the fancy, and scarcely consider it necessary to deny, what they do not feel seriously called on to believe. Some tables, constructed by the active observation of Dr. Latham, will serve to shew how futile is the popular creed in this particular instance.

It has been remarked by a keen observer of human nature, that a dogma, intended to exercise a wide and a permanent influence on mankind, must not appeal too clearly to the understanding. What all can comprehend and test by their common experience, implies no peculiar faith nor extraordinary merit in the believer.* We suppose that this principle will sufficiently account for the hold that such doctrines as that of critical days, have retained on the minds of physicians, from the time of Hippocrates to this. The few sober statements of Dr. Latham will complete the discomfiture of this ridiculous assumption.

Dr. Latham justly remarks that it is not always easy to say when a fever ends, or on what precise day it begins. The period of accession is, however, more commonly distinct than that of decline. A decisive phenomenon, or train of phenomena, as rigor, or sickness, or head-ache, may give precision to the former, whilst the latter has no such distinctive mark, and illness gradually merges in convalescence. This objection will appear so obvious and so striking to those of any experience, that we need not dwell upon it further. We must be content, in the generality of cases, to note that about such a time the patient began to get well, and it is on such data for calculating the termination of the malady, that the table we shall now introduce is founded.

Of 309 cases of fever, there were twelve of which the duration could not be satisfactorily determined. The reremaining 297 terminated on the days expressed in the table.

| Days of the Fever. | Number of Cases ending on each day. | Days of the Fever. | Number of Cases ending on each day. |
|---|---|---|---|
| 5 | 2 | 33 | 8 |
| 6 | 3 | 34 | 6 |
| 8 | 3 | 35 | 5 |
| 9 | 6 | 36 | 1 |
| 10 | 3 | 38 | 3 |
| 11 | 12 | 39 | 5 |
| 12 | 13 | 40 | 8 |
| 13 | 12 | 41 | 3 |
| 14 | 8 | 42 | 4 |
| 15 | 9 | 43 | 2 |
| 16 | 14 | 44 | 3 |
| 17 | 12 | 45 | 1 |
| 18 | 16 | 46 | 4 |

* Gibbon.

| | | | |
|---|---|---|---|
| 19 | 8 | 47 | 1 |
| 20 | 9 | 48 | 1 |
| 21 | 8 | 49 | 8 |
| 22 | 7 | 50 | 1 |
| 23 | 9 | 51 | 1 |
| 24 | 10 | 53 | 2 |
| 25 | 6 | 55 | 2 |
| 26 | 4 | 56 | 1 |
| 27 | 7 | 57 | 2 |
| 28 | 8 | 59 | 1 |
| 29 | 4 | 60 | 1 |
| 30 | 11 | 62 | 1 |
| 31 | 11 | 65 | 1 |
| 32 | 6 | | |

Dr. Latham makes the following observations on the foregoing list.

"Taking, then, the numbers exactly as they are represented in the table, it would appear that fewer cases terminated on the 14th and 21st day than on several days both prior and posterior to each. Out of two hundred and ninety-seven cases, eight only terminated on the 14th, and eight on the 21st.

For a fever to end before the 10th, or to be protracted beyond the 31st day, is unexpected and unusual. Thus a range of twenty-two days embraces the period within which the majority of cases come to their close; and, within this range, the table shews that there are twelve days (above half) more favourable to the termination of fever than either the 14th or 21st. Two equally favourable, and six only less so.

But let us allow a still larger latitude in seeking to know whether fevers are apt to come to their close *some time about* the 14th or 21st day. Let us take a range of three days, by joining each of these with the day immediately before and the day immediately after it; and then, adding together the number of cases which ended on the 13th, 14th, and 15th, and on the 20th, 21st, and 22d, let us see what proportion the sum bears to the number ending on any other three days taken consecutively.

Thus on the 13th, 14th, and 15th days, taken together, twenty-nine cases terminated; and on the 20th, 21st, and 22d, taken together, twenty-four cases terminated. These are our standards of comparison. But on the three days preceding our first standard of comparison, viz. the 10th, 11th, and 12th, twenty-eight cases terminated; and on the three days succeeding it, viz. the 16th, 17th, and 18th, forty-two cases. Again, on the three days preceding our second standard of comparison, viz. the 17th, 18th, and 19th, thirty-six cases terminated; and on the three days succeeding it, viz. the 23d, 24th, and 25th, twenty-five cases.

Finally, then, from the event of these two hundred and ninety-seven cases, no proof can be derived that there is any law of fevers inclining them to terminate upon one particular day more than another, or even *some time about* a particular day, whether it be the 14th or 21st, or any other day."

From all this it appears that the duration of fevers is indefinite. Dr. Latham has the merit of pointing out, in a methodic manner, what most men of sense and experience were sufficiently aware of.

Facts are so much superior to reasons, that any further consideration of the subject would be profitless and tiresome. But we cannot forbear from urging one point which would almost be sufficient of itself, to prove that fever can evince no determinate duration. At the time when the subtle Greeks gave laws to the progress and continuance of this affection, they were not aware of the organic lesions that attend, if they do not produce it. But the examination of the bodies of the dead, has shewn that ulcerations of the mucous membrane of the bowels, and organic alterations in the chest and in the head, are observed in a greater or less degree, in the majority of cases. Our knowledge of such alterations, precludes our belief in the possibility of their displaying particular days of decline and cure. An extensive ulcer of the aggregated glands of the ileum must cicatrize as other ulcers do, and the period of its cicatrization must be greatly modified by the nature of the treatment, the constitutional powers of the patient, and by other influences, frequent in occurrence, and powerful in operation.

### V. Two Cases of Laryngotomy.*

[Middlesex Hospital.]

Two interesting cases of this description have been related by Mr. Arnott. We will endeavour to present a succinct account of them.

Case 1.—*Poisoning by Nitric Acid—Laryngotomy—Death.*

J. R. æt. 13, accidentally swallowed some nitric acid, at 11 A. M. of May 13. The description given of the quantity taken was this—" he spat out a table spoonful, but must have swallowed some to enable him to draw breath: he had one gulp." He had breakfasted heartily at nine A. M. Water was given him immediately after the accident, and he was taken to a chemist's, where a solution of carbonate of soda was administered. Vomiting immediately took place, with great effervescence. He was then carried to the Middlesex Hospital, where calcined magnesia was given, by which the vomiting was encouraged. The tongue was swollen and of a citron appearance, the uvula and tonsils were enlarged, and there was great pain around the larynx and pharynx; the pulse was small and feeble. At 2 P. M. the sickness ceased, and he began to doze. At 4 P. M. there was slight coma; respiration was rather hoarse, and the voice was feeble. *Mixture of gum, honey, and chloride of lime, to moisten the mouth.* At 6 P. M. the coma had increased—face pale—pulse 120—surface, especially of the extremities, cold. At 10 P. M., the surface was of its natural temperature—face blanched—pulse fluttering—respiration more croupy and hoarse—lungs apparently imperfectly filled with air. *Fifteen leeches were applied to the throat.*

At midnight, Mr. Arnott was called. He found the boy lying in a state of apparent stupor; his face pale; the skin cool, almost cold; pulse frequent, feeble, and intermittent every fourth or fifth beat; his respiration was rough and difficult, the quantity of air taken into the lungs appearing to the ear applied to the chest to be but small. On being questioned, he raised his eye-lids, and spoke in a whisper. His replies were brief, quickly delivered, and apparently attended with increased suffering; for to other interrogations he answered by a look or slight nod. From the reason just mentioned, he could not readily open his mouth when requested to protrude his tongue, which he did not do. He was unable to swallow even a tea-spoonful of fluid. He had great pain all about the throat, from the front of which leech-bites were bleeding. On lateral compressure of the larynx, additional pain was felt. He had not vomited for some hours. There was no tenderness of the abdomen; and pressure on the region of the stomach gave no pain.

Under these circumstances Mr. Arnott determined to perform laryngotomy. He removed a piece of the crico-thyroid ligament. The sides of the wound in the parts external to the larynx were kept asunder by a bent loop of wire.

After the operation, relief appeared to be experienced, and at 10 A. M. of Tuesday there was less stupor—respiration frequent, but free, and exclusively carried on through the wound—pulse 120. *Calomel.* gr. iij. 2*dis horis.* The calomel passed in part, if not wholly, through the wound—respiration more frequent—no increase of secretion from trachea or bronchi. *Injections of beef tea per anum.* At half-past ten P. M. the patient died.

*Dissection.* " No discolouration or abrasion of the integument of the chin or lips. Teeth of usual white appearance. Upper surface of the tongue of a grey and citron colour, the former predominating at the tip and sides, the latter in the middle. The alteration in colour was seated in the cuticular covering of the part, which was separating, and hanging loose for some extent all round the edge of the tongue, and fairly detached from a part of its base, leaving the surface red, the papillæ bare, enlarged, and prominent. Membranous shreds hanging from both tonsils. The pharynx and upper third of the œsophagus were covered by a con-

* Med. Gaz. May, 18, 1833.

tinuous, grey-coloured, slightly lemon-tinted, membrane, which was corrugated and dry, and thrown into longitudinal plicæ, marked also by transverse lines. This membrane, the epithelium of the part, had become completely detached from the two lower thirds of the œsophagus, in which large portions of it remained loose, two or three small spots excepted, which were still covered with it; one of these close to the termination of the tube at the cardia. The surface from whence the epithelium had separated presented almost its natural smoothness, but was a little rosy in colour. On raising the lower edge of the portion of epithelium attached to the upper part of the œsophagus, the subjacent surface was red, which in some points had an arborescent character. The chorion of the œsophagus, especially of the upper part, was thicker than natural. The stomach internally, generally red, contained some yellowish brown, pappy, glairy matters. Its inner surface exhibited no softening or abrasion, or any unusual appearance, with the exception of one place, the size of a crown-piece, on the lesser curvature, on which were scattered a number of ochre coloured spots, the size of a pin's head, the colour in question being seated in the mucous follicles of the part, or gastric glands. The commencement of the duodenum had a healthy appearance.

The edges of the entrance of the glottis were extremely tumid; the surface at the same time corrugated; the colour lemon; but at two places, on opposite sides, of an orange colour. The epiglottis, of a brownish colour, was not merely corrugated, but shrunk in size, so that it could not be brought over the entrance of the glottis. The internal surface of the larynx, through its whole extent, from the opening of the glottis downwards, and the adjoining upper third of the trachea, was covered by a very thin delicate layer of lymph, which could be raised from the subjacent surface of the mucous membrane, which was here pale. Lower down, the mucous membrane of the trachea was of a deep red colour, and with no appearance of lymph. The bronchi also were red, but contained no diseased mucous secretion. The posterior parts of the lungs were loaded with blood."

The next case is one of a different complexion. In it laryngotomy was also performed, and with the same success.

Case 2. *Sore-throat — obstructed breathing—laryngotomy—death.*

J. S., aged between 40 and 50, was admitted into the hospital with fistula perinei and stricture of the urethra. Ten or eleven days after his admission, he was attacked with sore-throat, and on the evening of the same day he lost his voice. On the 19th day his countenance was anxious, the breathing hurried, accompanied by a gurgling, but not croupy; air was heard to pass through the larynx, with a sibilous noise occasionally, and freely into every part of the lungs. Pain was referred to the pharynx, and chiefly to the left side of it. None on pressing together the alæ of the thyroid cartilage. On the 20th day, he expressed relief from the application of leeches, but the respiration did not seem to be improved. During the night his breathing became worse, and the apothecary was called to him early in the morning; he applied twenty leeches.

At 10, a. m. Mr. Arnott was requested to see the patient. He found him apparently sinking under the symptoms above described. The breathing was laborious, attended with a rattling in the throat, the countenance slightly livid, but not anxious. The stethoscope to the chest discovered nothing, the noise in breathing preventing it. A blister having been applied, three days previously, over the front of the neck, (the surface of which was still sore,) rendered manual examination of the pharynx and larynx fruitless. Nothing amiss could be discovered in the posterior fauces by inspection.

Mr. A. determined to perform the operation, to which the patient willingly consented. Lymph was found effused at one place into the inter-muscular cellular substance. The breathing was temporarily relieved after the operation, but he subsequently sank, and died nine hours after its performance.

*Dissection.* "Coagulable lymph and pus were found effused at different parts into the cellular substance, all along the front of the larynx and trachea to the bottom of the neck, and some lymph into that of the anterior mediastinum. The pus and lymph were in greatest quantity on the left side of the upper part of the trachea.

In the pharynx, the entrance of the glottis was the seat of three ulcers, which occupied both edges of this aperture, without involving the mucous membrane of the larynx. These ulcers, however, burrowed deeply under this membrane, so that the probe could be passed in some directions, more especially forwards towards the angle of the thyroid cartilage to the extent of an inch. In the interjacent cellular substance of the larynx and pharynx, and amongst the muscles of the glottis, were in several places small abscesses; and on the left side, between the mucous membrane of the larynx and the thyroid cartilage, was one containing a teaspoonful of thick yellow matter.

The superior chordæ vocales might be a little thickened, but the mucous membrane of the larynx presented no other evidence of disease. There was some redness of the upper part of the trachea, increasing in intensity lower down. The bronchi were still more red, and their surface was covered with threads of puriform mucus. The lungs were loaded with blood. There was effusion into both sides of the chest of red coloured serum. The pleuræ were no way adherent."

Mr. Arnott appends a few remarks with which we do not altogether agree. He observes that,—

"The primary affection in this case was no doubt that of the throat; but did ulceration take place first, or were the ulcers the consequence of abscesses of the part breaking? It could not be ascertained from the friends that this man has been the subject of secondary syphilis. The effusion into the pleuræ, and the reddened, perhaps inflamed, bronchial membrane, were the result of laborious respiration from obstructed glottis. If the operation of laryngotomy had been performed sooner, both these consequences might possibly have been prevented; but how ought the burrowing ulcers of the pharynx and larynx, and the abscesses in the cellular substance of the latter, the chief cause of obstruction to the passage of air through the larynx, even if discovered, to have been treated?"

We do not perceive the connexion between the effusion in the pleuræ and the obstructed glottis, for no such obstruction is clearly mentioned in the notes of the dissection. We are informed that "the superior chordæ vocales *might* be a little thickened, while the mucous membrane of the larynx presented no other evidence of disease." If the ulcers "at the entrance of the glottis" occasioned an obstruction, it is not explicitly described.

But, allowing that there was obstruction, there is no necessity for resorting to this mechanical explanation of the pleural effusion and bronchitis. It is a fact which must be familiar to all accustomed to pathological examinations, that few persons die of inflammation of the cellular tissue about the neck, or indeed elsewhere, in whom traces of peripneumony or pleurisy, or both, are not discovered. A few days ago we assisted in examining the body of a patient, who died of diffuse inflammation of the mucous membrane of the stomach and bowels, and in whom there existed a condition of the thorax and its contents, similar to that observed in Mr. Arnott's patient.

We have seen many cases of inflammation of the cellular tissue, in the immediate neighbourhood of the pharynx and the larynx. In some, the seat of the disease was the tissue external to the mucous membrane of the larynx—in others, between the pharynx and the larynx—in others, anterior to the latter—in others, between the former and the spine. Wherever the inflammation was situated, it terminated in effusion of lymph or purulent matter, or both, produced nearly similar symptoms, and was followed by a similarly fatal result. We have said that the symptoms were similar; we might add that they were usually insidious. The patient appeared, in the first instance, to labour under common sore-throat, but the symptoms assumed a typhoid character, and were disproportioned to the obvious mischief. In some, the difficulty of swallowing preponderated; in others, that of respiration; but, in all, the depression was as remarkable as unaccountable. The first few cases were little understood, the patient dying when the medical attendant was unprepared for the event. But the fatal result of these cases, and the consequent disclosure of their real nature, induced us to form a more accurate and more gloomy prognosis in their successors. We may take an opportunity of bringing this subject before our readers in a more circumstantial manner.

We cannot suppose that laryngotomy will prove serviceable in this affection. The patient does not die from mechanical obstruction to the act of breathing, but rather from the grave impression that deep-seated suppuration always exercises on the system. He dies with typhoid symptoms, as he would if matter were locked up elsewhere. It may be said that incisions should be practised, and the lymph and pus discharged. But the seat of suppuration is neither certain, limited, nor, in general, accessible, and this circumstance, combined with the obscurity of the symptoms, must lead us to indulge in expectations the reverse of sanguine. Probably an acquaintance with the affection will enable us to do more in the way of prevention than of cure—by making us watch with great care all affections of the throat, and inducing us to employ active local depletion in suspicious cases. Calomel is not to be neglected.

We think that the profession are indebted to Mr. Arnott, for the candid and exact manner in which he has made his cases public. They form a very useful contribution to pathology.

---

### VI. Report of Cases, illustrating the Treatment of Gonorrhœa.

(Lock Hospital.)

[By H. J. Johnson, House-Surgeon.]

The treatment of gonorrhœa is confessedly unsatisfactory, often unsuccessful, and frequently empirical. The object of the present report is to display some of the varieties assumed by the disease, and the effects of remedies of different descriptions. As this paper is essentially a statement of facts, there is no necessity for adverting to questions that have occupied much attention, excited considerable and warm discussion, and have not been hitherto decided. I allude to the supposed affinity between gonorrhœa and syphilis, and the secondary symptoms that are said to succeed the former.

The number of cases of gonorrhœa treated at this hospital is necessarily great. They are seldom received into the house, and the circumstances of the patients affect the results, in much the same manner as in the ordinary routine of private practice. Medicines are taken irregularly and excesses are committed, as is commonly the case with patients not submitted to the restraints of hospitals, whatever their station in life may be.

Waiving all attempts at nosological distinctions, gonorrhœa may be divided, for practical purposes, into three leading varieties—gonorrhœa attended with much pain and inflammation—gonorrhœa attended with little inflammation—and gleet. But, independently of these affections, for separate affections they almost deserve to be considered, there are several symptoms or conditions of occasional occurrence, and of greater or less importance. Such are abscess of the cellular tissue of the penis—induration of the corpus spongiosum—lacunar inflammation and enlargement—pain in micturition, unaccompanied by discharge. When we add to this list the more immediate consequences of gonorrhœa—bubo, inflammation of the bladder, and hernia humoralis, I think it will be allowed that there is ample room for discrimination in the choice of remedies, and judgment in their application.

For my own part, I consider the hope of discovering a specific for gonorrhœa, as unphilosophical and futile. It is not a disease which presents and preserves a sufficient identity in various individuals, to be ever amenable to one plan of treatment. Means may be found of arresting the discharge, but this, so far from being always beneficial, is frequently quite the reverse. It may be urged that as gonorrhœa is a specific disease, a specific treatment may remove it. But I apprehend that the confidence in specifics is rapidly diminishing, and, if syphilis may be cited as an analogical instance, the specific tieatment of that complaint is one of the most complex problems in experimental medicine. I mean to imply, that though mercury is really a specific for syphilis, the treatment of that malady by means of its specific is extremely difficult, and requires much caution, judgment, and experience.

As gonorrhœa varies widely in its features in different individuals, it is necessary to consider the circumstances of the case, and to treat the particular assemblage of symptoms. The feature of most importance is the presence or absence of inflammation. In syphilis it is highly necessary to attend to this circumstance; in gonorrhœa it is equally or more so. If mercury is given in the former when much inflammatory action is present, it is probable that sloughing or phagedæna will ensue. If those medicines which check urethral discharge be administered in the latter, inflammation of the corpus spongiosum—of the bladder—or of the testicle, is apt to supervene.

I need scarcely enumerate the symptoms that constitute gonorrhœa attended with much inflammation. It is usually a first gonorrhœa, and there may or may not be general fever. The leading characteristics are, much scalding in the act of making water, painful erections at night, perhaps chordee, and discharge of thick purulent matter. But the latter is not a constant phenomenon, and frequently the secretion grows thin and watery, whilst the other inflammatory symptoms continue.

There are many ways of treating this inflammatory gonorrhœa—by cubebs or copaiba—by salines with antimony—by calomel and opium, with or without purgation—by colchicum—by diluents, with mucilage, nitre, and so forth.

There can be little question that cubebs and copaiba occasionally succeed, even in the inflammatory form of gonorrhœa; but there can also be little doubt that the experiment is one of a hazardous description. I will mention a few cases, in which this kind of treatment was adopted.

Case 1.—*Gonorrhœa treated by Cubebs—Hernia humoralis.**

Col. C—, who had frequently suffered from gonorrhœa previously, was again affected with it in the Winter of 1832. The discharge was thick and purulent, and there was considerable scalding in micturition. He consulted an eminent and an excellent surgeon, who prescribed the cubebs. I believe that the dose was raised to two drachms four times daily, or to more than that. The discharge was suddenly arrested, and hernia humoralis, with much inflammation of the cord, supervened. The Colonel was laid up with this complaint for two months, and it was necessary to put him slightly under the influence of mercury, before the affection was subdued. With the subsidence of the hernia humoralis, the discharge re-appeared, and was again yellow and thick, but unaccompanied by pain in the urethra. Subsequently he took small doses of cubebs and the discharge gradually ceased.

*Case* 2. About the time that I visited this patient, I was also in attendance on a young gentleman in the Regent's Park, situated under nearly similar circumstances. The gonorrhœa was combined with a good deal of scalding, although it was not his first. This gentleman was ordered cubebs by an

* The majority of the following cases occurred at the Lock Hospital. It will appear that some did not.

eminent surgeon. The discharge was suppressed, and acute inflammation of the testis and cord succeeded. There was great effusion into the tunica vaginalis of the testis, and suppuration was threatened. When the mouth was gently affected by mercury, this patient also got well.

I might mention many other instances of hernia humoralis, following the administration of cubebs, but this appears to be unnecessary.

CASE 3. *Discharge suppressed by Copaiba—suppurating Bubo.*

W. Andrews, a baker, æt. 29, admitted Sept. 7, 1833, under the care of Mr. Briggs. There was an inflamed and suppurating bubo in the right groin; pyrexia — loaded tongue—irregular bowels. The patient had been attacked one month previously with gonorrhœa. He took copaiba, and, a fortnight after the commencement of the gonorrhœa, the discharge ceased. The bubo then appeared.

The fact appears to be that where there is inflammation to any extent, stimulants or irritants of every description are commonly injurious. Cubebs or copaiba, an injection, a debauch, venereal excitement, or even exercise, aggravate the inflammatory action, which, confined no longer to the anterior part of the urethra, extends backwards to the bladder, and from that, along the mucous membrane of the vas deferens to the testis. Sometimes the urethral discharge is suppressed, but I think that commonly it is only diminished. Whether suppressed or not, it usually returns on the subsidence of the hernia humoralis.

CASE 4. *Acute Gonorrhœa—Inflammation of the Cellular Tissue of the Penis—Hernia Humoralis.*

A gentleman belonging to the University of Cambridge slept with a female in London with whom he was previously acquainted, and had frequent connection during the night. He stated that he experienced almost spasmodic and constant erections. Two days after this, he observed a slight urethral discharge, and tingling in the act of micturition. On the next, or the following day, I was requested to attend him.

The discharge was profuse and thick, the pain in making water great, and the cellular tissue of the penis was so infiltrated with serum, that the glans was almost buried in the mass of the paraphymosed prepuce. I immediately made scarifications with the lancet, and encouraged the bleeding by fomentations. I ordered calomel with active purgatives, and put the gentleman as speedily as possible under the nauseating influence of antimony, combined with colchicum. He was directed to drink large quantities of diluents. It was necessary to repeat the scarifications, and subsequently to apply leeches freely to the pubes, groins, and perinæum, indeed I contemplated bleeding from the arm to some extent, and in another similar case I would certainly adopt this measure. The inflammatory symptoms gradually abated, and after a fortnight or three weeks yellow discharge alone was left. I was about to prescribe some copaiba, when the gentleman imprudently took active exercise. The consequence was severe hernia humoralis, for which it was necessary to affect his mouth to a slight extent with mercury. He recovered from this in about five weeks. The urethral discharge continued, but it gradually subsided spontaneously, and in a month, or thereabouts, after the subsidence of the inflammation of the testis, this too had disappeared.

It is worthy of remark that, in this instance, there was no evidence of the female being affected with any discharge, and probably the excitement of the parts was exclusively the cause of the inflammation of the urethra.

CASE 5. *Gonorrhœa relieved by injections—exacerbation after travelling—chronic painful erections, &c.*

A commercial gentleman, of one of the midland counties, contracted a gonorrhœa, for which he went to an excellent friend of mine who resided in his town. He prescribed injections, with powders, I believe, of nitre and gum. Whatever was the treatment, the patient was greatly relieved, and would probably have soon been cured. Unfortunately he was obliged to travel to London, and the exertion of the journey, combined, perhaps, with some irregularities of diet, was productive of very injurious consequences. My friend had recommended this gentleman to my care, and he called on me on the morning succeeding his arrival in London.

He was now suffering extreme torture. The desire to make water was incessant—the pain in the act excruciating—the discharge abundant, but not very thick—the erections at night so painful that the patient jumped out of bed every quarter or half hour, and sat with the bare perinæum on the hearth. I tried diluents with mucilages, nitre, and alkalies, in vain. The following plan of treatment succeeded in relieving him.

℞. *Hyd. sub.* gr. v.—*Opii,* gr. j.—*Ant. tart.* gr. j. *M. ft. pil.* ij. *omni nocte sumend.*

*Haust. sennæ* c̄ *Magnes. sulph.* ℥ss.—*Vini colchici,* ʒss.—*Magnes. carb.* gr. xv. *omni mane.*

*Haust. salin.* c̄ *Vin. ant. tart.* ʒij.—*Vini colchici,* ♏xxv.—*Tinct. camph. c.* ʒj. *3tiis horis.*

The patient was so brought under the influence of antimony, that he could not, without difficulty, maintain the erect posture. Then, and not before, was the extraordinary suffering relieved; and, after he had been in town for a fortnight, he was able to quit it and return to the country. His mouth was slightly affected by the mercury—the discharge was abundant—there was no pain in micturition, but the painful erections were not quite gone. I have subsequently learnt that this patient again suffered from the latter symptom, and that it passed away on the application of a blister to the perinæum.

The preceding are instances of the acute form of gonorrhœa, and of the bad effects that frequently supervene on the use of stimulants, or the irritation occasioned by improper exercise.

I will now take the liberty of mentioning the plan of treatment that I have found productive of the most beneficial results. I think that, in the great majority of cases, the patient is benefited by the exhibition of a small quantity of mercury. There are two great indications in the treatment of gonorrhœa—first, to reduce the inflammation; secondly, to get rid of the discharge. If there is pain in making water, a few grains of mercury, combined with antimony, and, if necessary, with opium, should be given at bed-time for a few nights, and a purgative draught, combined with magnesia and a little wine of colchicum, should be taken on the ensuing morning. If the inflammatory symptoms run high, salines, with antimony, must be frequently exhibited during the day, and in every case the patient should drink large quantities of mucilaginous diluents, with nitrate of potass, if the scalding is excessive. It may possibly be necessary to employ general bleeding—more frequently it is advisable to apply leeches or cupping-glasses to the perinæum, and immerse the patient in a warm-bath. If, however, the inflammatory symptoms are not unequivocally great, there can be little question that saline medicines are rather injurious than useful. The discharge is apt to become all at once very thin, and a troublesome gleet succeeds. In these cases, we may substitute another combination with the mercury and the purgatives. It consists of the union of the powder of gum tragacanth, nitrate of potass, carbonate of potass, and Dover's powder. This frequently relieves the pain with great rapidity, and, when combined with mercury and aperients, has never been attended with unpleasant results.

The usual effect of the plan of treatment I have here sketched, is to relieve the pain in micturition, and diminish or remove the inflammatory symptoms. The discharge becomes, pari passu, more thin, and frequently more profuse. This is a point which requires much attention. It is highly necessary not to carry mercury or depletion beyond a certain extent. If the discharge becomes very thin, before these means are discontinued, it is much to be feared that interminable gleet will follow.

The difficulty is, to hit the moment when a change of treatment may be wisely ventured. If we are precipitate, the inflammatory symptoms return, and the scene is to be re-enacted;—if we are too late, the prospect of gleet is before us. I think I have observed that it is better to allow slight scalding to exist, when the change to which I have alluded is commenced; I mean that the antiphlogistic treatment should not be so pushed as to remove all pain in micturition. There should be a little, and a very little.

When the suffering is nearly gone, apparently just on the eve of departure, and when the discharge is profuse, and inclined to be thin, but yellow, I would recommend that cubebs, or copaiba should be given. My own predilection is in favour of copaiba, for I think that cubebs has succeeded best in those cases where there have never been inflammatory symptoms. Of this, I am far from certain, but such is my impression. The ordinary combination of copaiba with the spirit of nitrous æther is good, but that with the tinctures of kino or catechu is usually better. Cubebs may be given in combination with copaiba, or they may be exhibited in separate forms upon the same day. I do not dwell upon these points, because I am now advocating a principle of treatment, rather than weighing the advantages of detail.

Whether cubebs or copaiba be administered, the arrest of the discharge is commonly speedy and decisive. A few days will frequently be sufficient to effect an apparent cure. But this is too often delusive. The discharge is stopped, but merely for a time. After a few days it generally re-appears, watery, and a gleet. Now is the period for injections.

As soon as the discharge is manifestly yielding, or has yielded to cubebs or copaiba, I employ an injection of poppies and acetate of lead in the proportion of half a grain to the ounce. This should be used at night, and allowed to penetrate, without restraint, as far as the ordinary impulsion of the syringe will convey it. The copaiba or the cubebs should not be discontinued. In the course of a week or ten days the injection may be repeated more frequently, and its strength may be increased, whilst the other medicines are gradually abandoned.

I do not pretend that this plan of treatment will always be successful, but I think that it is so in the greater number of instances, and I believe that it is always safe. The watery discharge, observed only in the morning, continues for some time, and so long as it continues the injections must be used. There is another circumstance deserving of attention. After all apparent discharge has ceased the patient frequently complains of the lips of the orifice of the urethra being united in the morning by a sort of gum. The patient is liable at any time to a violent return of discharge. I have seen this occur in many instances. Whilst this appearance is observed, the injections should be persevered in, and mild aperient medicine occasionally taken.

I have said nothing of the diet of the patient during the inflammatory stage; it should of course be low. Afterwards a very spare diet appears to dispose to gleet. I have so frequently seen discharge kept up by wine and beer, and so frequently return on a resumption of these stimulants, that very great caution indeed must be exercised in their permission.

In the foregoing remarks I have not attempted to notice various plans of treatment, with which I am not unacquainted. I may without impropriety allude to the application of the nitrate of silver in the first instance—the use of a bougie introduced simply, or smeared with copaiba or with an ointment of catechu—strong injections of the nitrate of silver—large doses of cubebs. Perhaps I may be twitted with impertinence, for expressing my opinion that I am more disposed to admire than to imitate the courage of those who employ, and the confidence of those who submit to these methods. I cannot doubt, from the respectability of authority by which they have been sanctioned, that such measures have been productive of the happiest results. But the evidence of my senses has convinced me, that they are occasionally accompanied with risk, and that no precautions nor any judgement can in every instance guard against it. I fear that the cure of gonorrhœa is not, and never will be, effected "cito, tutè, ac jucundè." In my own person, I would prefer the tardy security of the second of these attributes, to the hazardous celerity of the first.

I will now relate some cases illustrative of the treatment of gonorrhœa above recommended.

CASE 6.—*Acute Gonorrhœa—Cure.*

Samuel Jacob, æt. 50, admitted as O. P. March 5th, 1833. Yellow discharge —much scalding—chordee. Slight nodule felt in the corpus spongiosum, posterior to the glans. Complaint for four weeks. Has done nothing.

*Hyd. sub.* gr. ij. *Opii,* gr. j. *Ant. Tart.* gr. ½. *M. omni nocte sumend. in noct. tres.*

*Liquoris potassæ,* ʒss. *Vini colchici,* ♏x. *Mucilags. acaciæ,* ʒj. *4tis horis è poculo Decocti hordei.*

April 20th. Soon after taking the medicine the discharge was diminished, and the pain and chordee disappeared. In the latter end of March the discharge ceased, and the complaint was apparently cured; but, having probably committed some excess, it returned after an absence of a fortnight. The return was last week. He has presented himself to-day, in consequence of this.

℞. *Confectionis cubebæ,* ʒij. *ter die.**

27th. Discharge again stopped.

May 12th. There has been no recurrence of the discharge since the last report. He has taken no medicine for the last ten days.

Dismissed cured.

This patient has come lately to shew himself. He has had no relapse.

* The composition of this is as follows. It was recommended to me by an intelligent young surgeon, Mr. Parsons. The disadvantage of it is, its extreme nastiness. But to return. The confection contains cubebs, copaiba, carbonate of magnesia, and confection of senna.

CASE 7.—*Acute Gonorrhœa—Cure.*

James Shanagham, æt. 18, a labourer, admitted Jan. 23d, 1833. Discharge and scalding, which have existed for five or six weeks.

*Liquoris potassæ,* ℥j. *Mucilaginis acaciæ,* ℥ij. *Aq. dist.* ℥iv. *Capt. coch.* ij. *magna 4ter die è poculo aquæ.*

*Haust. sennæ mane p. r. n.*

Feb. 19th. Discharge had disappeared for several days. He then left off the medicine, and probably committed an excess. He has now a relapse. Discharge yellow—return of scalding.

℞. *Bals. copaibæ,* ʒj. *Spir. æth. nit.* ♏xxv. *Tinct. catechu,* ʒss. *ter die.*

March 8th. Discharge gone for one week. No medicine for three or four days.

CASE 8.—*Acute Gonorrhœa—almost cured—Death from Cynanche Maligna—Examination of the Urethra.*

Thomas Ross, æt. 22, a servant out of place, admitted O. P. Aug. 23d, 1833. Thin yellow discharge—some pain in micturition—painful erections. Complaint for three weeks.

*Hyd. submur.* gr. v. *Ant. tart. Opii,* āā gr. ½. *M. omni nocte in* 3 *noctes.*

*Hs. salin.* c̄ *Mag. sulph.* ʒj. *bis die.*

26th. Complains of more pain. Obliged to lie about the streets at night, from poverty.

The patient was received into the house, and ordered a powder containing nitre, tragacanth, and Dover's powder, to be taken every four hours in barley-water. The pills, with two grains only of calomel, were repeated for two or three nights more.

In the course of a few days, the pain had almost ceased, the painful erections were gone, and the discharge was thinner and very profuse. He was ordered copaiba and catechu, and in less than a week very little discharge remained. He was now seized with angina maligna, in its most aggravated form, and in three days he died.

I examined the urethra. There was some inflammatory injection of the mucous membrane contained within the glans, and for an inch or thereabouts posterior to this. The rest of the canal was healthy.

CASE 9. *Acute Gonorrhœa—suppression of discharge, and relapse—cure.*

James Nally, æt. 30, a labourer, admitted O. P. July 26, 1833. Yellow discharge, with much pain. The discharge has existed for five days; the pain preceded it.

*Hyd. sub.*, gr. ij. *Opii*, gr. ½, *hâc nocte.*

*Haust. salin.* c̄ *Tr. hyos.* ♏x. *Vin. ant. tart.* ʒss. *Mag. sulph.* ʒj. 4*tis horis.*

Aug. 1st. Excoriation, from the discharge, on the glans and inner prepuce.

*Rep. Pil. o. n. in noctes tres. Lavatio tepida.*

5th. Excoriations healed. No pyrexia. Discharge whitish—still much pain in micturition, but no chordee.

*Omr. pil. et haust.*

*Pulv. ip. comp.*, gr. iij. *Pulv. trag. comp.* ʒj. *Magnes carb.* gr. xv. 4*tis horis è dec. hordei.*

*Haust. sennæ omni mane.*

9th. Scarcely any pain; what remains is immediately *after* making water—discharge much less. P.

23d. No pain—scarcely any discharge since the 11th. Has not applied since that day.

*Omr. meda.*

*Injectio bis die. Dec. papav.* c̄ *Plumb. acet.* gr. j. *ad* ℥j.

*Haust. sennæ, p. r. n.*

29th. Return of pain after using the injection—discharge aggravated.

*Omr. Injectio.*

*Hyd. sub.* gr. iij. *Opii* gr. ½ *hâc nocte.*

*Magnes sulph. sequente mane.*

Sept. 2d. Pain and discharge continue.

*Rep. Pulveres olim prescripti.*

*Magnes sulph. p. r. n.*

20th. No discharge nor pain for a week. Has taken no medicine for four days.

To take some powders, and re-apply if necessary.

CASE 10. *Very acute Gonorrhœa—apparent cure.*

W. Green, æt. 30, a policeman, admitted O. P., June 28th, 1833. Discharge yellow—excessive scalding—painful erections—complaint three or four days.

*Hyd. sub.* gr. ij. *hâc nocte.*

*Haust. sennæ omni mane in* 3 *vices.*

July 1. *Hs. salin.* c̄ *Vin. ant. t.* ♏xij. *Tr. hyos.* ʒss. *M. ter die.*

*Rep. Haust. senn.*

July 5. *Adde Hui. Mag. sulph.* ʒj. *et rep.* 4*tis horis.*

23d. Cuc. cruent. lumbis, ad ℥viij.

26th. These means have been productive of no relief. The urgency of the symptoms continues—the pain is intolerable. Probably too the nature of his duties as policeman, obliging him to be out and walking all night, has been mainly instrumental in aggravating the complaint.

*Om. Meda.*

*Pulv. ipec. comp.* gr. v. *Pulv. trag. c.* gr. xx. *Potass. nitratis*, gr. vj. 4*tis horis è dec. hordei.*

*Hyd. submur.* gr. ij. *Opii*, gr. j. *Ant. tart.* gr. ½. *omni nocte.*

*Magnes. sulph.* ℥ss. *p. r. n.*

Aug. 10th. The effect of these remedies has been remarkable. Scarcely any pain in micturition—painful erections continue, though less troublesome—still yellow discharge.

16th. *Omit. pil.*

*Emplast. cantharidis penis infer. parti.*

24th. The blister has finally removed all pain, and the painful erections are gone. Discharge less yellow, and not very thin.

*Bals. copaib. Sp. æth. nit.* āā ʒss. *bis die. Omr. alia.*

29th. Scarcely any discharge. Complains of itching within the urethra.

*Inject. papav.* c̄ *plumb. acet. omni nocte. Pulv. rhei* c̄ *magnes.* ℈j. *omni mane.*

Sept. 7th. Says he merely observes a little watery discharge, as the first thing in the morning.

*Rep. injectio nocte maneque. P.* c̄ *pulv.*

20th. Patient says he is well.

In the next case copaiba was given at the time chordee existed. The consequence was hernia humoralis.

CASE 11. *Discharge with Chordee—Hernia humoralis—cure.*

James Fowler, a pot-boy, æt. 23, admitted O. P. March 15th, 1833. Yellow discharge—not much scalding in micturition — considerable chordee. — Complaint for three weeks. Has done nothing for it.

℞. *Hyd. submur. Opii* āā gr. j. *omni nocte. Bals. copaib.* ʒj. *Sp. æth. nit., Liq. potass.* āā ʒss. *bis die.*

23d. Discharge much diminished. Chordee continues, as does the scalding. Right testis swollen, tender—epididymis indurated. Affection of testis for three days.

*Hirud.* xx. *scroto.*

*P.* c̄ *pil. omni nocte.*

*Haust. salin.* c̄ *Vin. ant. t.* ʒj. *Mag. sulph.* ʒj. 6*tis horis.*

*Omr. balsamum.*

26th. *Rep. hirud.* xij. *P.*

30th. Hernia humoralis cured. Still some chordee.

*Opii* gr. j. *Camph.* gr. v. *omni nocte.*
*Ung. hyd. fort.* c̄ *Camph. testi.*
*Mist. mag. sulph.* c̄ *mag. carb. bis die.*

April 10th. Chordee gone. Very little discharge.

20th. *Confec. cubebæ,* ʒij. *bis die.*

23d. Discharge has not appeared for two or three days.

May 4th. Discharge has never returned. Very slight induration of caput minus epididymis left.

The preceding cases appear to be sufficient to illustrate the treatment of gonorrhœa attended with much pain, or with chordee. They seem to prove satisfactorily the value of a small quantity of mercury, combined with opium and with antimony, in relieving the inflammatory symptoms. The cases also appear to shew that considerable pain will sometimes continue, when there is little or no pyrexia, and when it is probable that little inflammation exists. The remedy that exerts most influence on this condition, is, in my opinion, Dover's powder, with mucilage, and nitre, or magnesia. I have heard the liquor potassæ much recommended in the treatment of these symptoms. I have also heard nitre strenuously advocated. I have tried both separately, and I cannot but conclude that the combination I have mentioned is much superior to either.

Pain in the urethra (I am speaking of gonorrhœal cases) may present itself as a prominent symptom under three combinations of circumstances. It may accompany acute inflammatory symptoms, with which I have already alluded to it. It may attend purulent discharge, without other symptoms of much inflammation. Finally, it may exist as a solitary symptom, constituting, for practical purposes, a substantive affection. I have described the treatment which I have found most adapted to the two first varieties of pain, or rather, varieties of circumstances under which pain occurs. The last I shall reserve for separate consideration.

It will hardly be necessary to relate many cases of gonorrhœa attended with little inflammation. Cases of acute gonorrhœa are reduced to this state before they can be cured, and the instances which I have already given have shewn the treatment which appears to answer best. In order that the report may be more complete, I will subjoin a few.

It is this form of gonorrhœa which admits of the greatest variety of treatment. Some employ cubebs—some copaiba—some injections. All succeed, all occasionally fail. It certainly has appeared to me that success is more insured even in these instances by commencing the treatment with some calomel for a night or two, followed by aperient medicine. Then we may exhibit cubebs or copaiba, and when a decided impression is made on the discharge, injections may be usefully employed. I certainly would not recommend the use of an injection while pain is felt in the urethra. I must say that even in this milder form of gonorrhœa, I have seen much mischief from too stimulating treatment. I will now proceed to the cases.

CASE 12.—*Gonorrhœa with little inflammation—apparent cure.*

James Gillo, a cordwainer, æt. 24, admitted O. P. Aug. 17, 1833. Yellow discharge—some scalding—has had more. Complaint for eight days—has done nothing.

*Hyd. sub.* gr. iij. *hâc. et cras nocte.*

*Hs. salin.* c̄ *Mag. sul.* ʒj. *Vin. ant. t.* ℈j. 4*ter die.*

20th. More scalding—discharge thinner.

*Hyd. sub.* gr. v. *Opii. Ant. tart.* āā gr. j. *o. n. in noctes tres. P.* c̄ *haustu.*

24th. Less discharge and little scalding.

*Rep. pil. in noctes duas.*

26th. Discharge nearly gone — no scalding.

*Bals. copaib. Sp. æth. nit.* āā ʒss. *bis die—om. alia.*

30th. Much the same.

*Bals. copaib. Tinct. catechu,* āā ʒss *bis die.*
*Inject. dec. papav.* c̄ *Plumb. acet.* gr. j. *ad* ℥j. *omni nocte.*

Sept. 7th. Scarcely any discharge visible.

*Rep. injectio. ter die.*

*Pulv. rhei.* c̄ *Magnes.* ℈j. *o. n. Om. alia.*

14th. Thinks he has noticed a little water in the morning.

I directed the patient to continue the injection and return if the discharge should re-appear. I have not subsequently seen him.

CASE 13.—*Yellow discharge removed by injections.*

Robert Dixon, æt. 22, had had gonorrhœa for some time, succeeded by severe hernia humoralis. I believe that this was the effect of the injudicious exhibition of copaiba. At the time I first saw him, he had induration and pain in the body of the left testis and yellow discharge. I ordered him calomel and opium at night, salines with the sulphate of magnesia, and the mercurial ointment to the testicle. As these means were not productive of relief, I desired him to apply fourteen leeches to the testicle, and increased the quantity of opium at night. In the course of a fortnight from his first application, the testis was quite well; and discharge alone remained. For this I ordered the injection of decoction of poppies with acetate of lead, and the discharge disappeared.

CASE 14. W. Meadland, a tailor, æt. 21, admitted O. P. August 23, 1833. Discharge copious, thin, and yellow. No pain. Tendency to phymosis. Complaint has existed for two months, and there was much pain in the first instance. Has taken copaiba and the spir. æth. nit.

*Hyd. sub.* gr. v. *Opii,* gr. ½, *hâc et crast. nocte.*

*Injectio plumbi inter præput. et gland.*

*Bals. copaib.* c̄ *Tinct. catechu, bis die.*

26th. Phymosis much better. Discharge nearly gone. *Injiciat inject. in urethram, o. n.*

29th. No further discharge.

Sept. 9th. Discharge returned slightly yesterday, after a debauch with beer.

*Hyd. sub.* gr. iij. *Ext. col. c.* gr. v. *hâc nocte.*

*Magnes. sulph. cras.*

13th. Discharge scarcely perceptible.

*P.* c̄ *Inj. bis die.*

After this the discharge disappeared, but he was desired to continue the injection for a time, and to live soberly.

CASE 15. *Discharge without scalding —cured by injection.*

W. Jones, æt. 17, servant, had gonorrhœa followed by hernia humoralis. He came under my care as out patient on the 19th July. He had discharge of yellow colour, without scalding. There was some induration of the epididymis. I ordered him cathartic medicine, with colchicum, for a day or two, and then directed him to continue this in the morning, and use an acetate of lead injection twice daily.

Aug. 1st. Discharge has been arrested for two days.

23d. Has had a return of discharge two or three times. There has been none for three days.

29th. No return of discharge.

I have seen this patient since the last report. There has been no return of the discharge. For precaution's sake, he continued to use the injection occasionally.

CASE 16.—*Discharge cured by Copaiba and Injections.*

Thomas Errol, æt. 21, admitted O. P. July 6th, 1833. Yellow discharge for one fortnight. No pain in micturition. Has taken cubebs.

*Hyd. sub.* gr. v. *hâc nocte.*

*Haust. sennæ crâs.*

*Confect. cubebæ bis die.*

14th. No better. Has been drinking porter and spirits throughout. To discontinue these.

*Rep. Haust. sennæ et Pil.*

*Rep. Confect.* 4*ter die.*

24th. Discharge still thick and yellow.

*Omr. Cubeba.*
*Bals. copaib.* c̄ *Tr. catechu, bis die.*
*Rep. Pil. et Haust.*

Aug. 17th. "Very nearly well."

*P.* c̄ *Bals. semel die.*
*Inject. Dec. papav.* c̄ *Pl. acet. t. d.*

Sept. 1st. Cured.

I need not pursue the fatiguing detail of individual cases. They resemble each other in their leading features, and a narrative of experiments with cubebs or copaiba would not be attended with any interest. The cases I have mentioned are calculated to illustrate the treatment I have found most useful.

I would make a few remarks on the subject of cubebs. The confidence expressed in it by many surgeons has long occasioned astonishment on my part. I have seen it employed, and have employed it in a considerable number of cases; but it has seldom been my lot to find it productive of a cure. If the case be well chosen, it commonly stops the discharge; but the latter usually returns as soon as the cubebs is omitted, or even whilst it is continued. The cure is much more certain, if injections be combined with the cubebs as soon as the discharge assumes this fugacious character. Nearly the same observation appears to apply to the copaiba.

I have little to say on the subject of gleet. I believe that, in this affection, opposite methods will occasionally answer, but that, still more frequently, none will succeed. I have seen a gleet removed by blue-pill at night, and cathartic medicine on the following morning, continued for some little time. I have also seen a gleet removed by tonics—by turpentine—by injections—by copaiba. More frequently all these means have failed. Some surgeons appear to attach much importance to the use of the bougie. Whether my fortune has been worse than theirs, or whether I have selected the cases for this method with less discrimination, I will not venture to decide; but certain it is, that I never found the bougie succeed. We naturally attach a higher importance to what we see than to what we hear, and, actuated by this impression, I have little confidence in the employment of the instrument. If the cases I have witnessed lead to a preference of any one method, it is to small doses of mercury and cathartic medicine for a few days, succeeded by astringent or stimulating injections. Much as the solution of the nitrate of silver has been lauded, I have seldom seen it beneficial; but more good has been derived from injections of lead or zinc. It is not improbable that stimulating injections have been much abused in the treatment of both male and female discharges.

### Abscess of the Cellular Membrane of the Penis.

This is occasionally witnessed. I have seen three instances during this year. Two were in hospital patients ; one occurred in the person of a gentleman. As the latter was the one which I saw first, I will give it the priority.

Case 17.—*Gonorrhœa neglected—Abscess of the Penis—Chronic Induration of the Corpus Spongiosum.*

A gentleman contracted a gonorrhœa. Circumstances rendered it necessary for him to conceal the nature of his complaint, and he mixed in society, and drank wine. When I saw him the whole penis was considerably swollen from infiltration of the cellular tissue, particularly towards the free end of the prepuce, which was somewhat paraphymosed. The integuments were very red. Independent of the general swelling, I thought there was fluctuation, connected with the inferior surface of the corpus spongiosum, an inch, or thereabouts behind the glans. There was thick purulent discharge—the pain in micturition was great—the nocturnal erections distressing. The gentleman had for a few days been treated pretty actively with colchicum and mercurial purgatives.

I immediately opened the abscess, and two or three drachms of pus were discharged with relief. I ordered, if I remember rightly, leeches and fomentations, and calomel with antimony and opium were continued at night, while salines, with colchicum and sulphate of magnesia, were given during the day. The case was attended by a very intelligent friend of mine, and I saw the patient occasionally only.

The swelling gradually diminished, but was long ere it subsided. As soon as the declension of the subcutaneous effusion allowed a more accurate examination, it was found that the abscess was connected with the corpus spongiosum, or its investing membrane, though its essential seat was in the cellular tissue. The corpus spongiosum was generally indurated, from effusion of lymph.

As soon as the patient was enabled to leave his bed, which was three or four days, he was compelled to take exercise, and engage in occupations not calculated to hasten his recovery. It was an object of great importance that the nature of the illness should not be suspected, and fortunately, by much attention on his own part, as well as on that of his medical attendant, the affection, severe as it was, gave rise to no suspicions. The induration of the corpus spongiosum yielded very slowly, and the abscess reappeared once, if not twice. The treatment, consisted in slight affection of the gums by calomel and opium—saline purgatives with colchicum—mucilaginous drinks—alkaline aperients—and repeated blisters to the penis. It is now many months since I first saw this case, and it is only within the last fortnight that I deemed it prudent to attempt to check the discharge. Whilst chronic inflammation was going on in the corpus spongiosum, the discharge was looked on as a natural consequence, and I dreaded that copaiba or remedies of that description would have occasioned fresh abscesses, or hernia humoralis. A fortnight ago the induration of the corpus spongiosum was almost gone. A hard cord was felt in the site of the abscess, connecting the puckered skin with the corpus spongiosum. This cord was probably the contracted cellular cyst. There was no pain—no irritation. I prescribed copaiba and catechu in small doses. I saw the gentleman a few days ago. The medicine had been productive of no unpleasant result, and the discharge was rather diminished. I directed him to increase the dose.

Case 18.—*Abscess in the Cellular Tissue of Penis—discharge—cure.*

John Smith, æt. 20, admitted O. P. Feb. 19, 1833. Penis much swollen—integument red—prepuce disposed to be phymosed. Fluctuation felt on inferior surface of penis, where there is much pain on pressure. Discharge from within the urethra, with much scalding. Painful erections. Complaint for three weeks. Has been under a medical gentleman who treated him with copaiba.

I opened the abscess and let out half an ounce of pus. It had extended between the integument and corpus spongiosum, but it was not clear if the latter was implicated, or otherwise.

*Hyd. sub.* gr. v. *o. n. in noctes tres.*

*H. salin.* c̄ *Vin. colchici,* ♏ xxv. *Magnesiæ sulph.* ʒj. 6*tis. horis.*

*Cataplasma panis et fotus.*

The cavity of the abscess rapidly contracted, and the discharge became glairy. In about ten days it had ceased. The acute symptoms had passed away. After an interval, occupied by the employment of alkaline aperients, I ordered the tinctura lyttæ for the thin discharge which alone remained.* The dose was raised to thirty minims four times daily, when the abscess re-appeared, and there was return of irritation. The abscess was opened, and the patient again put upon aperient medicine. Again the abscess closed, and again the discharge grew thin. I prescribed copaiba. The copaiba produced an exanthema, with pains in the limbs, and pyrexia. The discharge was at this time almost arrested. Under aperients, the eruption passed away, and again the discharge returned. Thinking, that it might be kept up by some slight inflammation in the corpus spongiosum, which was rather hard in the site of the abscess, I ordered the following ointment to be applied to the inferior surface of the penis.

*Ung. hyd.* ℥j.—*Ant. tart.* ʒij. *Iodinæ* gr. x. *M. Applr. nocte maneque.*

This ointment usually occasions severe counter-irritation, of a character intermediate between pustule and vesicle. Two or three applications are commonly sufficient to produce the effect. In this instance it was very satisfactory. The discharge from the urethra diminished soon afterwards.

The patient was now put on copaiba and catechu. The discharge ceased, and on April 10th, he was dismissed cured.

The succeeding case differs in some respects from the preceding.

CASE 19.—*Circumscribed Abscess in the Cellular Tissue of the Penis—Seton employed.*

James Williams, a servant, admitted into the hospital, Aug. 11, under the care of Mr. Briggs.

Excessive induration on the dorsum of the penis, immediately behind the angle of reflection of the prepuce—induration globular, circumscribed, accompanied with a feeling of tension, as from fluid contained in a cyst. Bubo inclined to suppurate in either groin. Looks pale and out of health.

Had gonorrhœa two months ago. It passed away in about a week, under the administration of copaiba. Buboes immediately succeeded, and he has been ill from that time. The induration did not attract his attention until about a fortnight ago.

*Hyd. sub.* gr. ij. *hâc et crast. nocte.*
*Solut. Magnes. sulph.* c̄ *Mag. carb. omni alterno mane.*

26th. Swelling increased in size, tender to the touch, fluctuating more distinctly. It was opened through the inner prepuce, and two drachms of pus discharged. Bubo suppurating in two or three points in the right groin.

*Pil. Hyd.* gr. iij. *o. a. n.*

*Inf. ros.* c̄ *Mag. sul.* ʒj. *bis die.*

*Omr. alia.*

Sept. 12th. The abscess continuing to discharge, and the cavity not contracting, a probe was passed into it, through the opening in the inner prepuce, and the point cut upon through the outer prepuce. A seton-thread was then passed and secured, and the probe was withdrawn.

Sept. 20th. Cavity of the abscess almost obliterated. Seton-thread to be withdrawn in a few days.

* The tincturæ lyttæ has been much recommended in the treatment of gleet. I have tried it in many cases and succeeded in none.

### Induration of the Corpus Spongiosum Urethræ.

This is a frequent consequence of inflammatory gonorrhœa, especially if a stimulating treatment is adopted. It is a structural condition, of which chordee is a common symptom. Merely painful erections are seldom accompanied with distinct induration in the corpus spongiosum. In chordee the source of pain is usually the corpus spongiosum, somewhere between the glans and scrotum. Where there are erections without chordee, the patient refers his pain to the orifice of the urethra.

It does not seem necessary to relate any cases of induration of the corpus spongiosum. The affection is easily distinguished by examination with the finger, and, indeed, in every case of gonorrhœa, such an examination should be made. Lacunar enlargements and ordinary induration of the corpus spongiosum may exist independently of pain, and copaiba, or injections, might be used injuriously or without advantage, if the surgeon was unacquainted with the actual condition of the parts.

The treatment of inflammation and subsequent induration of the corpus spongiosum is so obvious, that it scarcely deserves observation here.—Leeches or cupping in the first instance, and subsequently blisters to the penis or perinæum. If the case has been neglected in its commencement, the induration is often extremely obstinate. I have seen a gentleman who applied eight or ten blisters to the penis, and was troubled with this affection for many months.

The application of the mercurial ointment has been much recommended and is greatly practised. In trivial cases it will answer well enough, but in those of any severity it has seemed to me extremely inefficient. Small quantities of mercury, purgatives, and bland diluents are serviceable auxiliaries, indeed they are almost indispensable. As a means of counter-irritation the ointment of mercury, tartarized antimony, and iodine, the formula of which was given in the last page, may be strongly recommended. Its action differs in some measure from that of a blister, and I have thought it rather more effectual.

Chordee is often treated in a very empirical manner. It appears to be considered a symptom of irritation rather than of inflammation, and narcotics, with camphor, are liberally prescribed. But true chordee can hardly exist without structural alteration of the corpus spongiosum, and that can scarcely be cured, frequently not benefited, by medicines of this description. The treatment of chordee, is, in general, the treatment of inflammation and induration of the corpus spongiosum, and on this nothing further need be said.

Painful erections are much more amenable to narcotic treatment. I think that the combination of Dover's powder with tragacanth, and magnesia, nitre, or rhubarb according to circumstances, is the most effectual remedy for this symptom. Occasionally opium and camphor, opium and tartar-emetic, conium morphia, &c. succeed. But even painful erections cannot always be treated in this manner. We must investigate their cause. I lately saw a gentleman who was greatly annoyed with this unpleasant occurrence. It appeared that he had always experienced an amatory, indeed a salacious disposition, and was in the habit of very frequent connexion. When suffering from gonorrhœa, he had of course desisted from gratifying his inclination, and the painful erections seemed, on a close consideration of the case, a consequence of unappeased desires. Opium had been tried, and had occasioned an aggravation of the complaint. I directed this gentleman to be cupped once or twice on the perinæum, to abstain from malt or vinous liquors, to eat very little meat, and to take some active purgative medicine every morning. This treatment was followed by an almost immediate disappearance of the erections.

### Enlargement of the Lacunar Glands of the Urethra

This is not uncommon, in connexion with inflammatory gonorrhœa. In that form of the complaint, the mucous membrane is always acutely inflamed, and it is not surprising that the lacunæ, and the corpus spongiosum should at times be implicated. The lacunæ, however, become the seat of inflammation and subsequent induration, in that form of gonorrhœa in which there is little inflammatory action. The most common situation of the affected lacuna, for commonly it is only one, is a little posterior to the glans. The induration, about the size of a pea, or larger, is generally felt from the under surface of the penis. Occasionally it is connected with the superior wall of the urethra, and is distinguished by firm pressure through the flaccid corpora cavernosa. In the first stage there is pain on pressure, in the second there is none.

This affection is commonly brought on, or if not brought on, it is increased and aggravated, by stimulating treatment. Cubebs, copaiba, or injections should never be given whilst the nodule remains. When attending the inflammatory stage of gonorrhœa, the treatment adapted for that stage is adapted for it, and under such treatment the lacunar enlargement usually recedes. Of this I have seen numerous examples. But when a nodule remains, unattended with inflammatory symptoms, the treatment I have found most useful, is mild aperients, abstinence from much animal food and from stimulants, and counter-irritation. I have seen some cases, in which attempts have been made to arrest the discharge during the presence of a lacunar nodule. Such attempts have done no good, but rather tended to a contrary result. It will only be necessary to relate one case illustrative of this affection.

CASE 20. *Discharge—Nodules connected with the Urethra — removed by counter-irritation.*

Robt. Mackenzie, æt. 32, a tailor, admitted O. P. July 26th, 1833. Yellow discharge. Little pain in micturition. Nodules felt connected with the corpus spongiosum urethræ. They are apparently chronic enlargements, with induration, of the lacunæ. The complaint has existed for two months. Has been taking cubebs, copaiba, &c. without any benefit.

*Hyd. sub.* gr. ij. *o. a. n.*

*Solut. cathart. o. a. m.*

*Pulv. rhei.* c̄ *Magnes.* ℈j. *bis die.*

Aug. 6th. P.

*Infricetur penis infer. parti Ung. hyd.* c̄ *Iodin. et Ant. tart.*

11th. Mouth slighly affected. Nodules much reduced in size—discharge much the same.

*Omr. pilulæ.*

19th. Discharge considerable, yellow, thin. Nodules very much reduced, but not quite gone.

*Dec. papav.* ℥j. *Plumb. acet.* gr. ½. *pro inject. o. n.*

*P.* c̄ *pulv. et solut. cathart.*

23d. Nodules no further diminished.

*Rep. unguentum.*

29th. Discharge rather increased.

*Hyd. sub.* gr. iij. *Opii,* gr. ½, *hâc nocte et crastinâ.*

Sept. 6th. Better.

*P.* c̄. *pulv. bis die et inject. o.n. Omr. alia.*

20th. Nodules have quite disappeared, since the last counter-irritation. The discharge alone remains. It is evident that the injection has not been serviceable to it. Ordered copaiba and catechu.

The object of the case was to display the treatment of the lacunar nodules. They were probably brought on by the perseverance in cubebs and copaiba in the first instance. The counter-irritation effected by the ointment in this case was excessively severe, yet six or seven weeks had elapsed before the nodules had quite disappeared. The unguentum hydrargyri is occasionally employed for their removal. It appears to me to be insufficient for that purpose; at least, it has been so in several instances which have fallen under my observation.

PAIN IN THE URETHRA, UNCONNECTED WITH DISCHARGE.

I mentioned, in some remarks upon pain in the urethra, that it seems to occur under three conditions—in inflammatory gonorrhœa—with thin discharge, unattended with inflammatory symptoms of any severity—and, finally, as a solitary symptom after others have disappeared. I am ignorant of the condition of the urethra which occasions it, and all that I can do is, to offer an account of some experiments, conducted with a view to ascertain the most successful method of treatment.

Case 21. *Pain in the Urethra—removed by Alterative Treatment.*

John Holliwell, æt. 52, admitted O. P. April 19th, 1833.

Very slight pain in micturition, referred to the inferior surface of the penis. It has existed for seven weeks. There was discharge in the first instance. For the last two days has again had slight and thin discharge.

*Confection. cubeb.* ʒij. *ter die.*

29th. Discharge almost gone—pain still troublesome.

*Emp. canth. penis infer. parti.*

*Rep. Confect. o. m. tantummodò.*

May 13th. Discharge has quite ceased for many days. Pain continues.

*Liquoris potass.* ♏xxv. *ter die.*

June 9. No better. Pain continues; it is acute, and felt always in the act of micturition—no feeling of hardness, nor of any thing unusual communicated on examination. Patient of a florid aspect—tongue moist, rather loaded.

*Pil. hyd.* gr. iij. *Pulv. ipec.* gr. j. *M. omni alt. nocte sumend.*

*Mist. cathart. o. a. m.*

July 5. Pain in micturition nearly, if not quite gone. Mouth has not been affected. Soon after this the patient discontinued his attendance, and I have reason to believe that he was quite well. This patient had enlarged prostate.

Case 22.—*Pain in micturition—not cured.*

John Robinson, æt. 19, admitted O. P. with pediculi, and pain in micturition, attended with little or no discharge. The pain had existed for eight or nine months.

The pediculi were speedily removed by a lotion of spirit of wine. The following means were then tried in succession, for the treatment of the pain in micturition. The ointment of tartar emetic—the ointment of mercury, iodine, and tartar emetic—aperients of the sulphate, and carbonate of magnesia twice daily—blister to the penis—salines with sulphate of magnesia—and the liquor potassæ.

The pain was somewhat mitigated, but far from cured, by these measures, and, shortly after the commencement of the exhibition of liquor potassæ, the patient discontinued his attendance.

Case 23.—*Sores—Pain in Micturition—cure.*

Mordecai Barnett, æt. 43, admitted O. P. April 20, 1833.

Two elevated sores on left side of penis, near scrotum. They appear to have been originally pustules. Has been for some time under treatment ineffectually.

*Pil. hyd.* gr. v. *omni nocte.*

*Infus. ros.* c̄ *Mag. sulph.* ʒj. *o. n.*

May 4. Sores healed—elevated cicatrices remain. Complains of much pain in the urethra after micturition. No urethral discharge.

*Emplast. canthar. penis.*

12th. Still complains of much pain in the urethra, now felt, however, before micturition.

*Liquor. potass.* ʒiss. *Pot. nitrat.* ʒss. *Muc. acaciæ,* ʒss. *Tr. hyos.* ℥iv. *Aq.* Oj. *M. bibat. vicibus partitis quotidie.*

*P.* c̄ *pilulis.*

*Magnes. sulph.* ℥ss. *o. m.*

May 28th. Scarcely any vestige of sores. The pain in micturition, though relieved, continues. Mouth has been gently affected.

*Omr. pil.—P.* c̄ *mist. et mag. sulph.*

June 15th. Has lately had connexion with his wife after a long absence from her. Believes that she has no venereal complaint. A fresh pustule has appeared on the dorsum of the penis, with induration of its basis.

*Cataplasma panis. P.*

18th. Sore better.

*H. sal.* c̄ *mag. sul. t. d.*

22d. Sore larger—yellowish on its surface—its base more hard and tumid. Looks pale.

*Dec. cinch.* c̄ *sod. carb. bis die.*

*Haust. senn. altern. mane.*

29th. Sore spreading sloughily towards the pubes, and assuming the herpetic character.

*Dec. sars. c.* Oss. *Ext.* ʒj. *quotid.*

*P.* c̄ *h. senn.—Lot. nig.* c̄ *catap. pane.*

July 6th. Sore has ceased to spread.

20th. Sore just healed. During the time occupied by the preceding reports, the pain in micturition has continued, with little alteration. There is and has been no urethral discharge whatever, nor has any alteration in the urethra or corpus spongiosum been perceptible. A bougie has been passed. It aggravated the pain.—*Cuc. cruent. perineo. ad* ℥x.

26th. Pain nearly gone since the cupping. Sore healed.

After this, the urethral pain quite disappeared. The patient attended as O. P. until the 17th of August, when he was finally dismissed, quite well. He has shewn himself subsequently, and has had no return of his complaint.

These are all the cases of this affection which it is at present in my power to relate. It must be owned that the treatment is not satisfactory, which may perhaps be owing, in some degree, to our ignorance of the actual condition of the urethra. The plan that appears to promise most is moderate purging with mercurial alteratives, and local depletion or counter-irritation. Attention to the history of the local complaint, as well as to the actual condition of the patient—the state of his digestive organs—his occupation—diet, &c. may perhaps be useful in assisting the choice of remedies.

